# Click-based amplification: designed to facilitate various target labelling modes with ultralow background amplification†

**DOI:** 10.1039/d1cb00002k

**Published:** 2021-03-20

**Authors:** Jinyi Bai, Fusheng Guo, Mengyao Li, Yulong Li, Xiaoguang Lei

**Affiliations:** Beijing National Laboratory for Molecular Sciences, State Key Laboratory of Natural and Biomimetic Drugs, Key Laboratory of Bioorganic Chemistry and Molecular Engineering of Ministry of Education, Department of Chemical Biology, College of Chemistry and Molecular Engineering, Synthetic and Functional Biomolecules Center, Peking University Beijing 100871 People's Republic of China xglei@pku.edu.cn; Peking-Tsinghua Center for Life Sciences, Academy for Advanced Interdisciplinary Studies, Peking University Beijing 100871 People's Republic of China yulongli@pku.edu.cn; State Key Laboratory of Membrane Biology, Peking University School of Life Sciences Beijing China; PKU-IDG/McGovern Institute for Brain Research Beijing China; Chinese Institute for Brain Research Beijing China

## Abstract

We here describe a fluorescent signal amplification method termed “Click-based amplification” that can be well integrated with various click-labelling modes, including chemical labelling, genetic incorporation and covalent inhibitor probe mediated target labelling. Picolyl azide (pAz) was used as a functional group of a streptavidin-based amplifier to enhance the efficiency of click chemistry. Click-based amplification provided 3.0–12.7 fold amplification on fixed HeLa cells with different click-labelling modes. Click-based amplification has proven to be superior to tyramide signal amplification (TSA) in view of its low nonspecific amplification and high signal-to-noise ratio. Moreover, in terms of the challenging signal amplification of tissue specimens, Click-based amplification successfully achieved remarkable fluorescence enhancement on intestinal tissue slices of afatinib-N_3_ treated mice, which provided direct evidence of the presence of afatinib-N_3_ in the intestinal tissues and helped in revealing the off-target toxicity of afatinib. Collectively, these results illustrate that Click-based amplification could serve as a promising method for bioimaging studies.

## Abstract 2

We describe a signal amplification method termed “Click-based amplification” that can be well integrated with various click-labelling modes, including chemical labelling, genetic incorporation and covalent inhibitor probe mediated target labelling.

## Graphical abstract

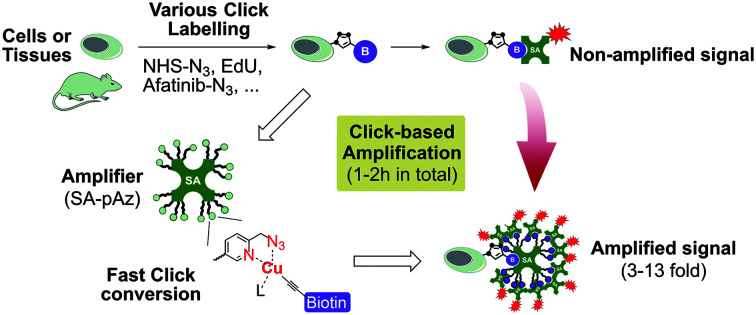

## Introduction

Signal amplification is widely demanded in various applications including western blotting, ELISA, microscopy and clinical diagnosis. Different signal amplification strategies have been developed to meet various requirements in practical applications. Tyramide signal amplification (TSA) uses horseradish peroxidase (HRP) conjugated streptavidin and antibodies as reporters to amplify signals by catalysing labelled tyramide substrate deposition.^1^ Hybridization chain reaction (HCR) utilizes one initiator DNA strand to trigger a cascade of hybridization events of two stable species of DNA hairpins, providing a linear amplification.^2^ Furthermore, to combine hybridization-based signal amplification methods with antibody-based immunostaining, immunosignal hybridization chain reaction (isHCR)^3^ and immunostaining with signal amplification by exchange reaction (Immuno-SABER)^4^ were developed and successfully used to amplify protein targets in tissues.

On the other hand, the abundance levels of biomolecules vary greatly. For example, the abundance of proteins in mammalian cells varies by at least seven orders of magnitude (about 10^1^ to 10^8^ copies per cell).^5^ To amplify targets with a wider range of abundance, some controllable amplification methods are developed using stepwise amplification strategies. Click-amplifying FISH (clampFISH) is a signal amplification method using iterative rounds of hybridization and click-locking to amplify RNA and DNA targets with high specificity and high amplification efficiency.^6^ Fluorescent signal amplification *via* cyclic staining of target molecules (FRACTAL) can amplify the signal intensity of immunofluorescence staining more than nine-fold *via* simple cyclic staining of secondary antibodies.^7^ Multi-cycle amplification provides a successive growth of signal intensity, but the background amplification and the signal-to-noise ratio should be carefully examined during the amplification process. More importantly, most of the current amplification methods share the same idea that target recognition depends on primary antibodies and hybridization reactions, resulting in the target range that is limited to proteins and nucleic acids.

Non-proteinaceous biomolecules, including glycans and lipids, also play important roles in various biological processes, but most of them show weak immunogenicity.^8^ Chemical biologists have developed a click-labelling platform to visualize various biomolecules:^9–11^ install unique functional groups into target molecules, then ligate visual tags and other reporters *via* bioorthogonal reactions. However, signal amplification methods tailored for click-labelling strategies are seriously inadequate. Carell and co-workers reported a remarkable dendrimer-based signal amplification using click chemistry, and achieved fluorescence amplification for EdU-labelled DNA *in situ* with low background amplification.^12^ However, other click-labelling and more complex biological systems were not tested.

Herein, we report a signal amplification method, Click-based amplification, that can amplify various click-labelling modes, including chemical labelling, genetic incorporation and covalent inhibitor probe mediated target labelling. Click-based amplification enhanced the fluorescence signal of fixed HeLa cells by 3.0–12.7 fold without introducing obvious nonspecific amplification. Finally, Click-based amplification was used to amplify the signal of mouse intestinal tissue specimens, providing direct evidence of drug distribution in animal tissues.

## Results and discussion

### Click-based amplification design

**Fig. 1:**
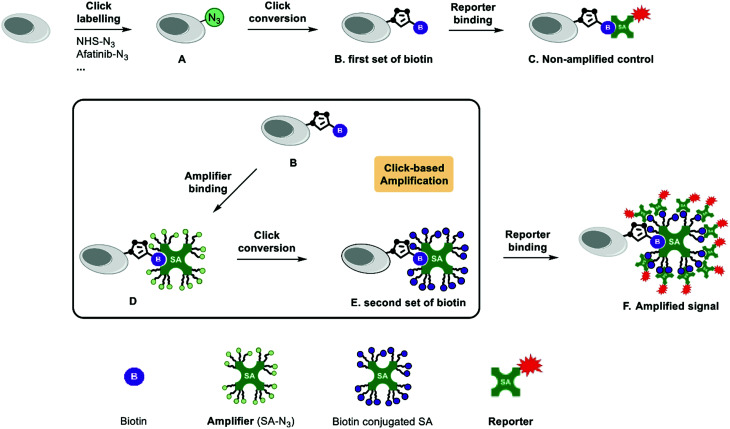
Schematic representation of a Click-based amplification procedure with streptavidin-N_3_. (A) The target of interest is labelled with the azide. (B) The first set of biotin tags are introduced *via* click reaction. (C) Non-amplified control: streptavidin-AF647 binds to the biotin on the target for imaging. (D) Amplifier binding step. Streptavidin-N_3_ binds to the biotin tags on the target and introduces multiple azide groups. (E) Click conversion step. A second set of biotin is introduced *via* click reaction. (F) Amplified signal: the additional biotin tags recruit more streptavidin-AF647 and result in enhanced fluorescence.

**Fig. 2:**
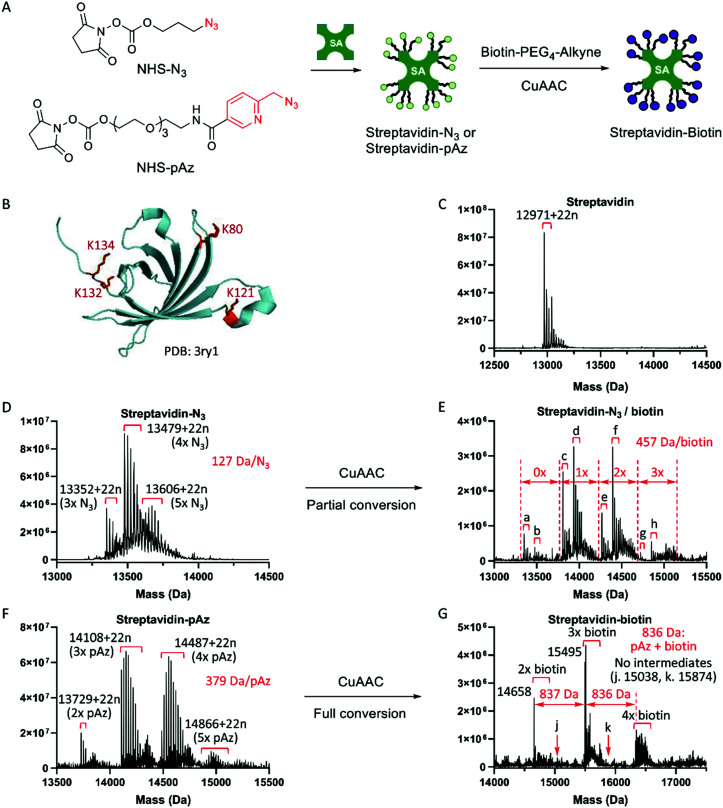
Streptavidin-pAz showed much higher click conversion efficiency than streptavidin-N_3_ in solution. (A) Reactions of synthesis of two types of amplifiers and Click conversion. Streptavidin was modified with NHS-N_3_ or NHS-pAz, generating two types of amplifiers streptavidin-N_3_ and streptavidin-pAz. (B) Crystal structure of a streptavidin monomer, including 4 lysine residues. (C) The deconvoluted ESI-TOF mass spectrum of the streptavidin monomer (mSA). Molecular weight (MW): 12 971 + 22*n*. Δ*m* = 22 should be Na^+^. (D) Streptavidin modified with NHS-N_3_. The numbers of N_3_ groups on streptavidin were calculated to be 3–5. (E) Partial conversion of streptavidin-N_3_ in (D) *via* CuAAC with biotin-PEG_4_-alkyne. MWs of a–h: 13 352 + 22n (3× N_3_, no biotin), 13 479 + 22n (4× N_3_, no biotin), 13 809 + 22n (2× N_3_, 1× biotin), 13 936 + 22n (3× N_3_, 1× biotin), 14 266 + 22n (1× N_3_, 2× biotin), 14 393 + 22n (2× N_3_, 2× biotin), 14 723 + 22n (no N_3_, 3× biotin), and 14 850 + 22n (1× N_3_, 3× biotin). (F) Streptavidin modified with NHS-pAz. The numbers of pAz groups on streptavidin were calculated to be 2–5. (G) Full conversion of streptavidin-pAz in (F) *via* CuAAC with biotin-PEG_4_-alkyne. MWs: 14 658 (no pAz, 2× biotin), 15 495 and 15 509 (no pAz, 3× biotin), and 16 345 (no pAz, 4× biotin). No intermediates, MWs: 15 038 (1× pAz, 2× biotin) and 15 874 (1× pAz, 3× biotin).

The schematic representation of Click-based amplification in this study is shown in Fig. 1. The amplification procedure includes two steps: (1) amplifier binding, azide functionalized streptavidin (amplifier) binds to the biotin tag on the target (Fig. 1D); and (2) click conversion, a second set of biotin tags are introduced onto the target *via* click reaction (Fig. 1E). The number of biotin tags on the target will increase after this two-step amplification process (Fig. 1B and E), since there are 16 azide groups on the amplifier in theory after saturated modification of streptavidin lysine residues with NHS-N_3_. Streptavidin is a tetramer protein, and every monomer contains 4 lysine residues: K80, K121, K132 and K134 (Fig. 2B, PDB: 3ry1).^13^ The saturated modification of the limited number of lysine residues can result in a relatively homogenous amplifier and thus a moderate amplification efficiency. Multi-cycle amplification is feasible in design, and the procedure is shown in Fig. S1A (ESI†). However, multi-cycle amplification may decrease the signal-to-noise ratio, since the amplified fluorescence of the vehicle control may increase faster than that with click-labelling. Therefore, one cycle of amplification is recommended to achieve a high signal-to-noise ratio and time and cost reduction.

### Optimization of Click-based amplification

#### Choosing biotin-PEG_4_-alkyne as the click converter

Click reaction was utilized to convert the azide groups on the amplifier to biotin. Two types of widely-used click reactions are tested in the Click-based amplification paradigm: copper(i)-catalyzed azide–alkyne cycloaddition (CuAAC)^14^ and strain-promoted azide–alkyne cycloaddition (SPAAC).^15^ We carried out Click-based amplification with the CuAAC-type converter biotin-PEG_4_-alkyne and the SPAAC-type converter biotin-PEG_2_-DIBO in fixed HeLa cells (Fig. S1A, ESI†). The confocal imaging and fluorescence quantification results are shown in Fig. S1B and C (ESI†). There was no significant increase of nonspecific fluorescence for biotin-PEG_4_-alkyne (Fig. S1C, ESI†), while biotin-PEG_2_-DIBO introduced high nonspecific binding (Fig. S1D, ESI,† No Amp., 1.8-fold) and high nonspecific amplification (Fig. S1D, ESI,† 7.6-fold for Amp.×2, 8.7-fold for Amp.×4). DIBO was reported to show a relatively higher azide-independent labelling of proteins than the terminal alkyne *in vitro*.^16^ Hence the CuAAC-type converter biotin-PEG_4_-alkyne was chosen for Click-based amplification.

#### Selection of picolyl azide (pAz) as a functional group of the streptavidin-based amplifier

The number of azide groups on the amplifier and the efficiency of subsequent click conversion are the key to achieving signal amplification (Fig. 2A). We first quantified the number of azide groups on streptavidin after saturated modification with NHS-N_3_ using an ESI-TOF mass spectrometer, and the increased molecular weight showed that 3–5 azide groups were covalently linked on the streptavidin monomer (Fig. 2D), indicating that a close-to-saturation modification of streptavidin was achieved, and that a small portion of the N-terminal α-amine was also modified. However, there were only 1.53 biotin groups conjugated onto the streptavidin monomer after click reaction in solution (Fig. 2E and Fig. S2A, ESI†). The low efficiency would reduce the overall amplification ratio. Picolyl azide (pAz) was found to accelerate the CuAAC reaction more than ten-fold due to the chelation effect with the copper(i) catalyst.^17,18^ We then synthesized a new modification reagent, NHS-pAz (Fig. S10, ESI†), and carried out the saturated modification and click conversion using the same protocol. The results showed that 2–5 pAz groups were conjugated to the streptavidin monomer (Fig. 2F), and the average number of pAz groups was 3.48 (Fig. S2B, ESI†), comparable to the number of N_3_ groups in streptavidin-N_3_ (Fig. 2D). But most importantly, all the pAz groups on streptavidin reacted with biotin-PEG_4_-alkyne in solution (Fig. 2G), and the average number of conjugated biotin groups was 3.16 (Fig. S2C, ESI†). Hence the copper-chelating azide (pAz) was chosen for Click-based amplification.

### Click-based amplification for NHS-N_3_ labelling in HeLa cells

**Fig. 3:**
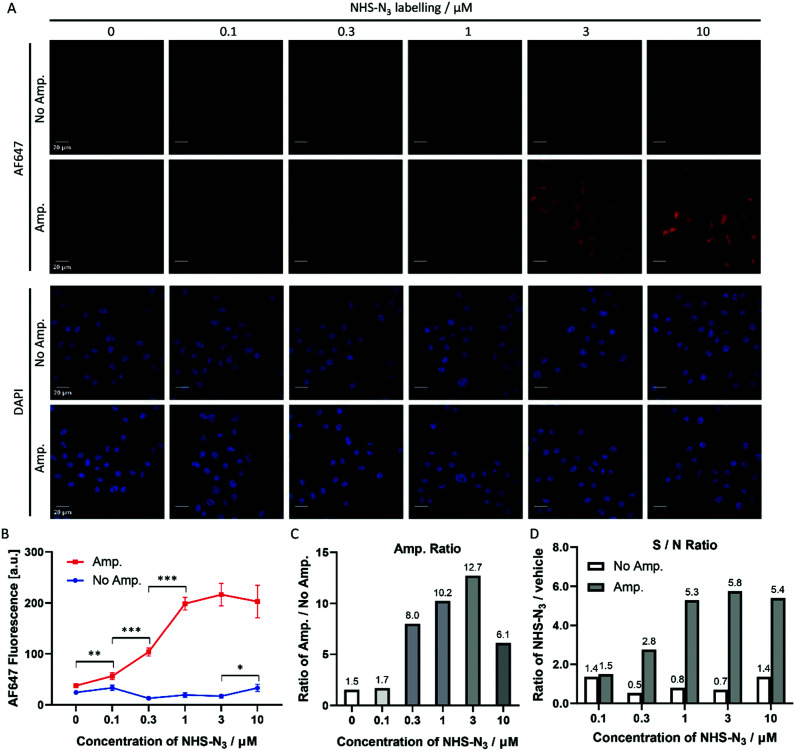
Click-based amplification on fixed HeLa cells with NHS-N_3_ labelling. (A) Confocal imaging of fixed HeLa cells without and with Click-based amplification (No Amp. and Amp.). HeLa cells were treated with a concentration gradient of NHS-N_3_ from 0.1 μM to 10 μM. DAPI stained the nuclei of the HeLa cells. Scale bars, 20 μm. (B) Quantification of the cellular AF647 fluorescence intensities in (A) with ImageJ. The red line represents the fluorescence intensity of HeLa cells with Click-based amplification (Amp.), and each data point is the average fluorescence intensity of 20 cells chosen randomly from the microscopy imaging. The blue line represents the fluorescence intensity of HeLa cells without Click-based amplification (No Amp.). Error bar: the standard error (SE). (C) Amplification ratios (Amp./No Amp.). (D) Signal-to-noise ratios (NHS-N_3_/vehicle). ***p < 0.001, **p < 0.01, *p < 0.05.

NHS-N_3_ globally labelled the biomolecules containing primary amino groups in HeLa cells, *i.e.* proteins and lipids. To determine the suitable range of labelling concentrations for Click-based amplification, HeLa cells were treated with a gradient of NHS-N_3_ from 0.1 μM to 10 μM. The cellular AF647 fluorescence intensity of confocal imaging (Fig. 3A) was measured with ImageJ. In the amplification group (Fig. 3B, Amp.), HeLa cells treated with 0.1 μM NHS-N_3_ showed significantly increased cellular fluorescent signals compared to the vehicle group, while in the group without amplification (Fig. 3B, No Amp.), HeLa cells treated with 10 μM NHS-N_3_ began to show a labelling-dependent fluorescence enhancement. Click-based amplification therefore improved the detection sensitivity by at least 100-fold. To quantify the amplification efficiency, the amplification ratio was defined as the ratio of the cellular fluorescence with Click-based amplification to that without amplification. Click-based amplification obtained fluorescence amplification by 6.1–12.7 fold in HeLa cells treated with 0.3–10 μM NHS-N_3_ (Fig. 3C). The signal-to-noise ratios of cellular fluorescence were also improved with Click-based amplification (Fig. 3D).

### Click-based amplification for EdU labelling in HeLa cells

**Fig. 4:**
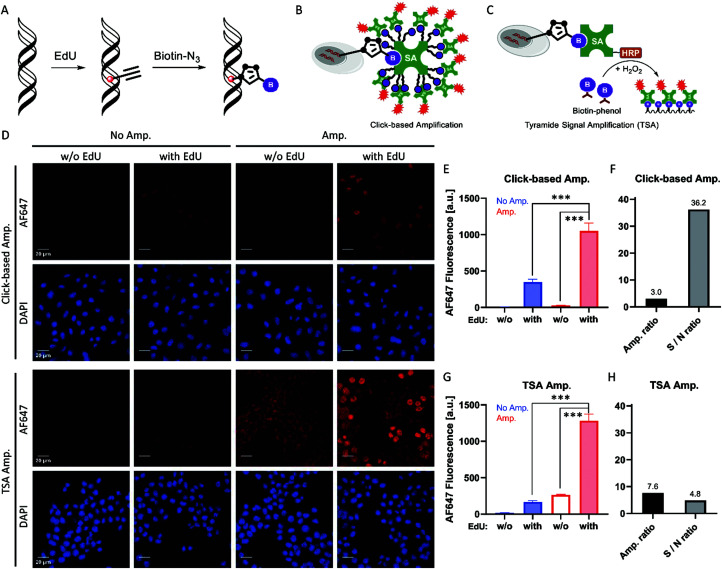
Click-based amplification achieved a much higher signal-to-noise ratio than TSA on fixed HeLa cells with EdU labelling. (A) Scheme of DNA labelled with EdU and the click conversion of EdU to biotin. (B) Scheme of Click-based amplification. (C) Scheme of TSA. (D) Confocal imaging of fixed HeLa cells with Click-based amplification and TSA. HeLa cells were treated with 10 μM EdU in DMEM at 37 °C overnight. DAPI stained the nuclei of the HeLa cells. Scale bars, 20 μm. (E) Quantification of the cellular AF647 fluorescence intensities in (D) with Click-based amplification. For the groups w/o EdU labelling, all the cell nuclei were dark, and 20 cells were chosen randomly to measure the average fluorescence intensity. For the groups with EdU labelling, all the lighted nuclei were measured to determine the average fluorescence intensity. (F) Amplification ratios (Amp./No Amp.) and signal-to-noise ratios (EdU/vehicle) with Click-based amplification. (G) Quantification of the cellular AF647 fluorescence intensities in (D) with TSA amplification. For the groups w/o EdU labelling, 20 cells were chosen randomly to measure the average fluorescence intensity. For the groups with EdU labelling, all the lighted nuclei were measured to determine the average fluorescence intensity. (H) Amplification ratios (Amp./No Amp.) and signal-to-noise ratios (EdU/vehicle) with TSA. ***p < 0.001.

5-Ethynyl-2′-deoxyuridine (EdU) has been used to be genetically incorporated into DNA during cell division. This approach labels the newly synthesized DNA with the alkyne group,^10^ which can be converted to biotin tags *via* click reaction with biotin-N_3_ (Fig. 4A). The nuclear positioning makes EdU labelling a suitable system to evaluate the fidelity of Click-based amplification. The confocal imaging results showed that Click-based amplification enhances the nuclear fluorescence signal but not that of cytoplasm (Fig. 4D). The amplification ratio of EdU labelling in HeLa cells was 3.0-fold (Fig. 4E and F). The relatively low amplification ratio may result from the highly crowded nuclear surroundings.

To evaluate the potential application of Click-based amplification, we compared our results with the widely used tyramide signal amplification (TSA). TSA can be readily integrated with various biotin labelling methods (Fig. 4C). We performed TSA for EdU labelling in HeLa cells. The results showed that TSA not only obtained a relatively higher amplification ratio, 7.6-fold (Fig. 4G and H), but also introduced obvious nonspecific amplification, since strong fluorescence was observed in the cytoplasm (Fig. 4D). The nonspecific amplification of TSA increased 29% when the concentration of SA-HRP increased from 1 : 1000 to 1 : 300 (Fig. S7, ESI†). Finally, the signal-to-noise ratio of Click-based amplification was 36.2, while that of TSA was only 4.8, mainly resulting from the high nonspecific binding of HRP.

HRP is a glycoprotein with nine potential *N*-glycosylation sites, and the carbohydrate content reaches about 20% (w/w).^19^ The high glycosylation of HRP may mediate relatively high nonspecific binding and high background amplification.^20^ In contrast, streptavidin has no glycosylation sites, which helps in explaining the ultralow background amplification of Click-based amplification.

### Click-based amplification for covalent inhibitor probe mediated protein labelling in HeLa cells

**Fig. 5:**
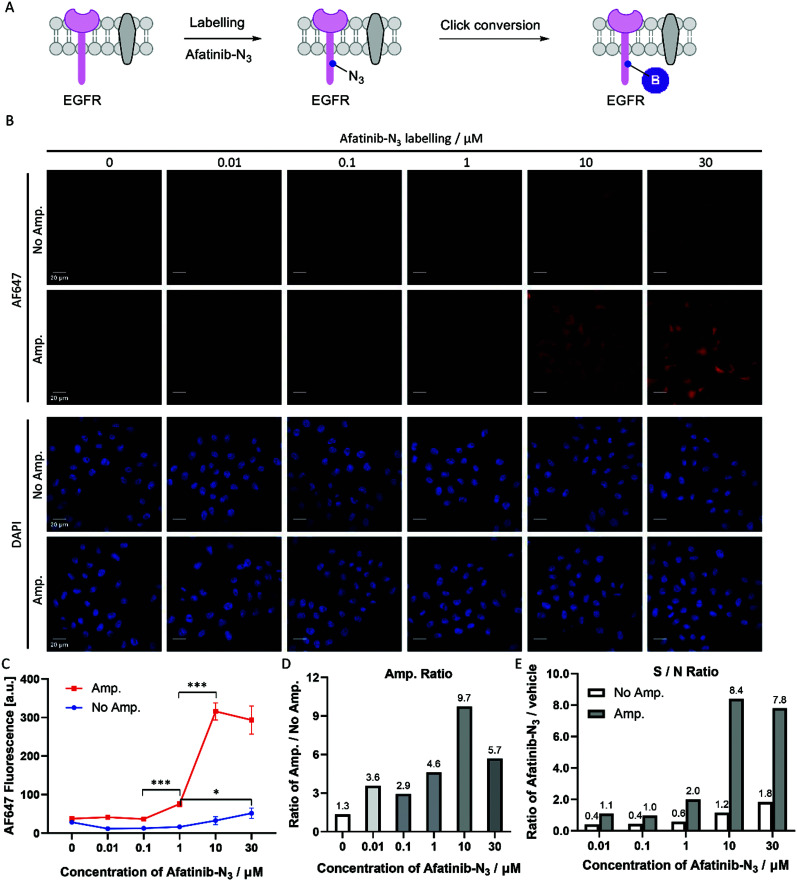
Click-based amplification on fixed HeLa cells with afatinib-N_3_ labelling. (A) Scheme of afatinib-N_3_ labelling on EGFR protein and the click conversion of N_3_ to biotin. (B) Confocal imaging of fixed HeLa cells without and with Click-based amplification (No Amp. and Amp.). HeLa cells were treated with a concentration gradient of afatinib-N3 from 0.01 μM to 30 μM in DMEM at 37 °C for 1 h. DAPI stained the nuclei of the HeLa cells. Scale bars, 20 μm. (C) Quantification of the cellular AF647 fluorescence intensities in (B) with ImageJ. The red line represents the fluorescence intensity of HeLa cells with Click-based amplification (Amp.), and each data point is the average fluorescence intensity of 20 cells chosen randomly from the microscopy imaging. The blue line represents the fluorescence intensity of HeLa cells without Click-based amplification (No Amp.). Error bar: the standard error (SE). (D) Amplification ratios (Amp./No Amp.). (E) Signal-to-noise ratios (afatinib-N_3_/vehicle). ***p < 0.001, *p < 0.05.

Covalent inhibitors are compounds that are designed to form a covalent bond with their specific molecular target. Today, there are more than 50 approved drugs that act as covalent inhibitors targeting kinases, RAS proteins, cathepsin, caspases and other enzymes.^21,22^ Afatinib is a covalent inhibitor of EGFR with an IC_50_ value of 0.5 nM, and was approved by the FDA and EMA in 2013 for the treatment of patients with advanced non-small cell lung cancer (NSCLC).^23^ HeLa cells express high levels of EGFR, and afatinib inhibits the growth of HeLa cells with an IC_50_ value of 6.8 μM.^24^ We designed and synthesized an azide functionalized probe, afatinib-N_3_, by replacing one *N*-methyl with a propyl azide group in our previously published paper.^25^ HeLa cells were treated with a gradient of afatinib-N_3_ from 0.01 μM to 30 μM (Fig. 5B). In the amplification group (Fig. 5C, Amp.), HeLa cells treated with 1 μM afatinib-N_3_ began to show a significant fluorescence increase, while in the group without amplification (Fig. 5C, No Amp.), HeLa cells treated with 30 μM afatinib-N_3_ began to show a labelling-dependent fluorescence increase. Click-based amplification therefore improved the detection sensitivity by at least 30-fold. In view of the IC_50_ value of 6.8 μM inhibiting HeLa cell growth, Click-based amplification serves as a more suitable imaging tool to reveal the relationship between the cellular location and biological function of small molecule inhibitors. Besides, Click-based amplification obtained fluorescence amplification by 4.6–9.7 fold in fixed HeLa cells treated with 1–30 μM afatinib-N_3_ (Fig. 5D). The signal-to-noise ratios of cellular fluorescence were also improved by Click-based amplification (Fig. 5E).

### Click-based amplification for drug distribution in mouse intestinal sections

**Fig. 6:**
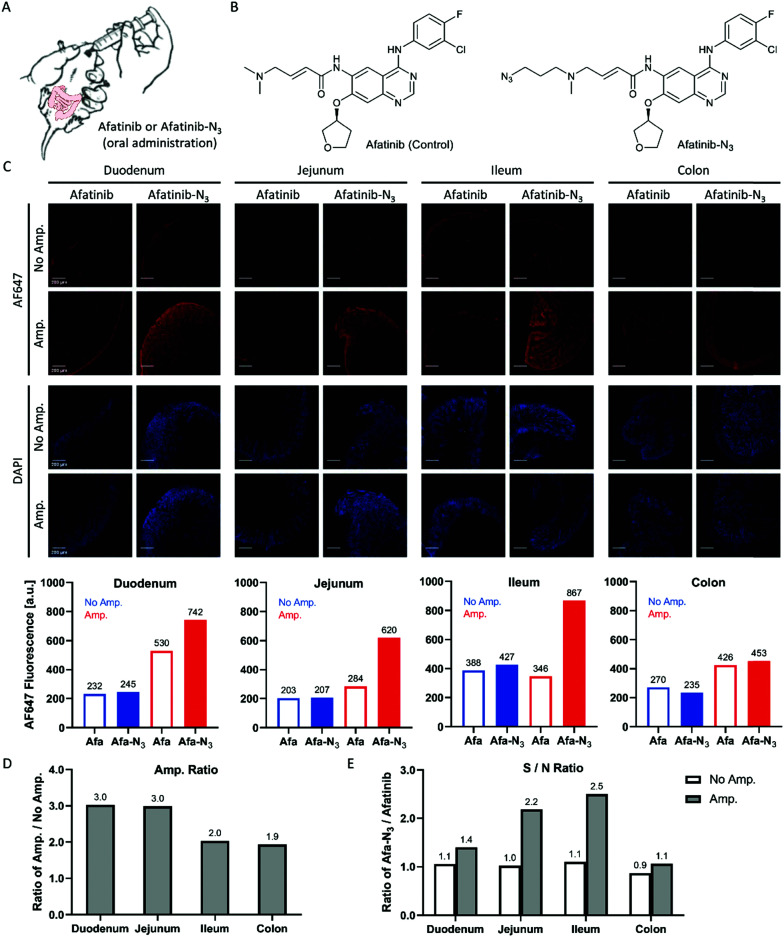
Click-based amplification on fixed intestinal sections of mice treated with afatinib-N_3_. (A) Scheme of P.O. treatment of mice with afatinib and afatinib-N_3_. (B) Structure of afatinib (control) and afatinib-N_3_. (C) Top: confocal imaging of fixed intestinal sections without and with Click-based amplification. The mice were orally administered with afatinib (10.0 mg kg^−1^) or afatinib-N_3_ (11.4 mg kg^−1^, equal mol), and dissection was carried out 24 h later. DAPI stained the nuclei of the intestinal cells. Scale bars, 200 μm. Bottom: Quantification of tissue fluorescence intensities. The whole specimen was circled and measured for the mean fluorescence intensity with ImageJ. Blue column, tissue fluorescence intensity without Click-based amplification. Red column, tissue fluorescence intensity with Click-based amplification. (D) Amplification ratios (Amp./No Amp.). (E) Signal-to-noise ratios (afatinib-N_3_/afatinib).

Afatinib frequently brings side effects to patients such as diarrhea, acneiform eruption, mouth sores, paronychia and dry mouth during clinical use.^26,27^ The signalling pathway modulations of afatinib have been uncovered,^28,29^ but there are currently few methods that can detect the localization of afatinib in tissues and provide direct evidence of the participation of afatinib in the occurrence of side effects. Y. Yamamoto *et al*. developed an immunohistochemistry (IHC) protocol using a specific anti-afatinib antibody and HRP/DAB (3,3′-diamino-benzidine) staining system to detect afatinib–protein conjugates in fixed rat intestines and skin tissues.^30^ Here, we treated mice with the afatinib-N_3_ probe (Fig. 6A and B) and carried out Click-based amplification in fixed intestinal sections from the duodenum, jejunum, ileum and colon. The fluorescence signals of the sections from the duodenum, jejunum and ileum were enhanced with Click-based amplification by 2–3 fold (Fig. 6C and D). The signal-to-noise ratios were also increased significantly with Click-based amplification for the sections from the jejunum and ileum (Fig. 6E). The sections from the colon showed the weakest fluorescence signals among these intestinal specimens, which suggested that the distribution of afatinib-N_3_ in the colon was low. Taken together, Click-based amplification may serve as a useful tool for detecting drug distribution in tissues.

## Conclusions

In summary, Click-based amplification has been demonstrated to amplify various click-labelling systems with ultralow background amplification. The streptavidin-based amplifier is a minimalist design: it has a moderate number of functional groups, is homogenous, is of small size, is cheap on the market, and can be stored as ready-to-use aliquots in a refrigerator. Picolyl azide (pAz) was used as the functional group of the amplifier to accelerate the click conversion.

Click-based amplification could be well integrated with various click-labelling modes, and provided fluorescence amplification for NHS-N_3_, EdU and afatinib-N_3_ labelling by 3.0–12.7 fold in fixed HeLa cells. Compared with the widely used TSA, Click-based amplification introduced very low nonspecific amplification when used in cell imaging, and gave a higher signal-to-noise ratio. Signal amplification of tissue specimens is challenging. Click-based amplification achieved a moderate signal amplification and improved the signal-to-noise ratio of afatinib-N_3_ labelling in fixed mouse intestinal sections. Tissue imaging with the drug probe would provide direct visualization of drug distribution, and could be complementary to target protein imaging. Collectively, we could expect that this newly developed Click-based amplification method will find broad application in biomedical research.

## Experimental methods

### General organic synthesis

The organic reactions involved in this work were carried out in flasks containing an argon atmosphere, which were sealed with a rubber stopper. All chemical reagents purchased from commercial suppliers were used without further purification. The reaction solvents were ultra-dry solvents containing molecular sieves, and transferred with injection syringes. TLC and LC-MS were used to monitor reactions, and silica gel column chromatography purifications were carried out to separate the intermediates and products. Products were characterized with ^1^H-NMR (400 MHz), ^13^C-NMR (101 MHz) and HRMS.

### Proteins and chemicals

Streptavidin (bs-0437P, Bioss), Streptavidin-AF647 (bs-0437P-AF647, Bioss), SA-HRP (B110053-0100, Diamond), Biotin-XX Tyramide (A8012-10, APExBIO), a BCA Protein Assay Kit (PT0001, Leagene), ProLong Gold Antifade Mountant with DAPI (P36941, Invitrogen), EdU (ST067, Beyotime), afatinib (BD210970, Bide Pharmatech), Biotin-PEG_4_-alkyne (#764213, Sigma), and BTTAA (BDJ-4, Confluore) were used.

### Synthesis of streptavidin-based amplifiers

#### Streptavidin-N_3_

To a solution of streptavidin (2 mg mL^−1^) in PBS, a DMSO stock solution of NHS-N_3_ was added in one portion and the final working concentration of NHS-N_3_ was 6.9 mM (200 eq.). The reaction mixture was rolling over at room temperature for 2 h, then loaded onto a HiTrap desalting column (29-0486-84, GE). Protein fractions were collected and combined. The concentration of streptavidin-N_3_ was quantified with the BCA Protein Assay Kit (streptavidin as the standard protein). The protein solution was divided into 10 μL aliquots and stored in a −80 °C refrigerator as a ready-to-use reagent.

#### Streptavidin-pAz

To a solution of streptavidin (2 mg mL^−1^) in PBS, a DMSO stock solution of NHS-pAz was added in one portion and the final working concentration of NHS-pAz was 5.0 mM (145 eq.). The reaction mixture was rolling over at room temperature for 4 h, then loaded onto a HiTrap desalting column. The subsequent operations were the same as those for streptavidin-N_3_.

### Click conversion of the amplifiers in solution

To a solution of streptavidin-N_3_ (0.21 mg mL^−1^ by BCA) or streptavidin-pAz (0.17 mg mL^−1^ by BCA) in PBS, 0.5 mM biotin-PEG_4_-alkyne, 0.5 mM CuSO_4_, 1 mM BTTAA and 2.5 mM sodium ascorbate were added. The reaction mixture was rolling over at room temperature for 2 h, then another 2.5 mM sodium ascorbate was added. The reaction was maintained for another 1 h. The protein product was purified with a HiTrap desalting column, using Milli-Q purified water as the eluent. After freeze drying, the protein powder was redissolved with water containing 0.3% formic acid. The protein sample was analysed with an ESI-TOF mass spectrometer. The molecular weight was obtained after deconvolution, and the click conversion efficiency was calculated based on the integration of MS peaks.

### Cell culture and labelling

#### Cell culture

HeLa cells were maintained in DMEM (Gibco) supplemented with 10% (v/v) fetal bovine serum (Gibco) and 100 IU of penicillin–streptomycin (Gibco) in a 37 °C incubator with 5% CO_2_. For click-labelling and fluorescence imaging, HeLa cells were resuspended at a concentration of 100 000 cells per mL in DMEM and seeded into a 24-well plate at 0.5 mL per well. A 12 mm glass coverslip was placed at the bottom of each well to allow HeLa cell adhesion.

#### NHS-N_3_ labelling and click conversion

HeLa cells adhered to the glass coverslip in the 24-well plate overnight. The medium was removed, and cells were washed with ice-cooled PBS twice, then placed on ice. NHS-N_3_ was diluted with ice-cooled PBS to a concentration gradient from 0.1 μM to 10 μM, and added into the 24-well plate. HeLa cells were treated with NHS-N_3_ on ice for 30 min, and washed with PBS twice. Cell fixation with 4% PFA was carried out at room temperature for 10 min, then quenched with 50 mM NH_4_Cl/glycine in PBS. The click conversion of N_3_ labelling was performed with 10 μM biotin-PEG_4_-alkyne, 100 μM CuSO_4_, 200 μM BTTAA and 2.5 mM sodium ascorbate in PBS at room temperature for 30 min. Cells were washed with PBS five times after click reaction.

#### EdU labeling and click conversion

After adhesion to the glass coverslip, HeLa cells were treated with 10 μM EdU in DMEM overnight. The medium was removed, and cells were washed with PBS twice. Cell fixation with 4% PFA was carried out at room temperature for 10 min, then quenched with 50 mM NH_4_Cl/glycine in PBS. Cells were permeabilized with 0.2% Triton X-100 in PBS for 10 min, and washed with PBS twice. The click conversion of alkyne labelling was performed with 10 μM biotin-N_3_, 100 μM CuSO_4_ and 2.5 mM sodium ascorbate in PBS at room temperature for 30 min. BTTAA was absent in the click reaction, because it slowed down the click conversion of EdU in HeLa cells (Fig. S6, ESI†). Cells were washed with PBS five times after the click reaction.

#### Afatinib-N_3_ labelling and click conversion

HeLa cells adhered to the glass coverslip at the bottom of the 24-well plate overnight. A series of 500× DMSO stock solutions (1 μL) of afatinib-N_3_ were added to the cell culture medium directly and mixed well. The final working concentration gradient of afatinib-N_3_ was from 0.01 μM to 30 μM. HeLa cells were maintained in an incubator for 1 h. Then the medium was removed, and cells were washed with PBS twice. Cell fixation and click conversion were carried out the same way as in NHS-N_3_ labelling.

### Click-based amplification workflow

#### Streptavidin-pAz binding

To the HeLa cells with biotin labelling, 0.5 μg mL^−1^ of streptavidin-pAz in PBS was added. The cells were maintained at room temperature for 40 min, then washed with PBS twice. The unoccupied biotin-binding pockets of streptavidin were blocked with 0.5 mM biotin in PBS at room temperature for 5 min, and HeLa cells were washed with PBS twice.

#### Click conversion

The click conversion of pAz groups on the target was performed with 10 μM biotin-PEG_4_-alkyne, 100 μM CuSO_4_, 200 μM BTTAA and 2.5 mM sodium ascorbate in PBS at room temperature for 30 min. Cells were washed with PBS five times after click reaction.

#### Streptavidin-AF647 binding

To the HeLa cells with biotin labelling, 1.0 μg mL^−1^ of streptavidin-AF647 in PBS was added. The cells were maintained at room temperature for 40 min, then washed with PBS five times.

#### Anti-fade mounting

ProLong Gold Antifade Mountant with DAPI (10 μL) was pipetted onto a microscope slide. The glass coverslip with HeLa cells was taken out from the 24-well plate with fine-tipped tweezers and inverted onto the antifade mountant droplet (the side with cells faced down). The sample was kept in the shade at room temperature for 24 h for solidification.

### Tyramide signal amplification (TSA) workflow

TSA was carried out following the indication of the TSA Plus Biotin Kit (PerkinElmer, NEL749A001KT). TNT wash buffer contains 0.1 M Tris–HCl (pH 7.5), 0.15 M NaCl and 0.05% Tween 20.

#### SA-HRP binding

The HeLa cells with biotin labelling were blocked with TNB buffer (TNT buffer + 0.5% BSA) for 30 min. SA-HRP (1 : 1000) in TNB buffer was added to HeLa cells and incubated for 30 min. Cells were washed with TNT buffer three times. (BSA was used as the blocking reagent in TNB buffer, because the recommended blocking reagent FP1020 by PerkinElmer was not commercially available at that moment.)

#### TSA amplification

HeLa cells were incubated with an amplification working solution containing 1 μg mL^−1^ of Biotin-XX-Tyramide (BXXP) and 1 mM H_2_O_2_ in 0.1 M borate buffer (pH 8.5) for 10 min, and washed with TNT buffer three times.

#### Streptavidin-AF647 binding

The HeLa cells with biotin labelling were blocked with TNB buffer for 20 min. 1.0 μg mL^−1^ of streptavidin-AF647 in TNB buffer was added to HeLa cells and incubated for 30 min. Cells were washed with TNT buffer three times.

Anti-fade mounting was performed the same way as that in Click-based amplification.

### Animals and afatinib/afatinib-N_3_ treatment

All animals in this study were bought from a company (Charles River, China) and acclimated for one week before experimental use. All the animals were housed in the barrier facility of the laboratory animal center with 12 hours of light and 12 hours of darkness, and all experiments were approved by the Institutional Animal Use and Care Committee. Male C57BL/6 (8 week old) mice were divided into two groups: the afatinib group (*n* = 3) and afatinib-N_3_ group (*n* = 3). Both compounds were dissolved in a solution containing 10% DMSO and 40% 2-hydroxypropyl-beta-cyclodextrin in water, and orally administered to the mice (10.0 mg kg^−1^ and 11.4 mg kg^−1^ respectively). Dissection was performed 24 h later. Duodenum, jejunum, ileum and colon specimens were collected and embedded in an optimal cutting temperature compound (OCT compound). Cryostat sectioning was carried out by cutting 8 μm thick sections in a slicer at −20 °C. The intestinal tissue sections were frozen at −80 °C before use.

### Click-based amplification of mouse intestinal sections

An ImmEdge™ Pen (Vector Laboratories, H-4000) was used for drawing a water-repellent barrier around the tissue section that was mounted on a microscope slide. The tissue specimen was washed with PBS twice, fixed with 4% PFA for 10 min, then quenched with 50 mM NH_4_Cl/glycine in PBS. The click conversion of N_3_ labelling was performed with 10 μM biotin-PEG_4_-alkyne, 100 μM CuSO_4_, 200 μM BTTAA and 2.5 mM sodium ascorbate in PBS at room temperature for 30 min. The tissue specimen was washed with PBS five times after click reaction.

#### The Click-based amplification workflow for fixed tissue sections was the same as that mentioned above except for the anti-fade mounting

ProLong Gold Antifade Mountant with DAPI (10 μL) was pipetted onto the tissue specimen directly, then an 18 mm × 18 mm glass coverslip was placed on the tissue specimen. The sample was kept in the shade at room temperature for 24 h for solidification.

### Confocal imaging and fluorescence quantification

#### Confocal imaging method

Cells on 12 mm coverslips were imaged using an inverted TiE A1 confocal microscope (Nikon) equipped with a 60×/1.40 NA oil-immersion objective, a 405 nm blue-violet laser (coherent) and a 641 nm laser (coherent). Blue fluorescence and red fluorescence were collected using a 450/50 nm emission filter and a 700/75 nm emission filter, respectively. Tissue sections were imaged using the same confocal microscope but equipped with a 10×/0.45 NA objective.

#### Cellular fluorescence quantification with ImageJ

Single cells were circled along the membrane edge and measured to calculate the mean fluorescence intensity, a similar field without any cells was circled and measured to determine the background fluorescence, and the difference was defined as the cellular fluorescence intensity. Generally, 20 cells were chosen randomly and their fluorescence intensities were averaged to get the mean cellular fluorescence and the SE value, and an example is given in Fig. S4 (ESI†). For HeLa cells with EdU labelling, all the lighted nuclei were circled and measured to calculate the average nucleus fluorescence intensity.

## Ethical statement

All animal procedures were performed in accordance with the Guidelines for the Care and Use of Laboratory Animals of Peking University and approved by the Animal Ethics Committee of Peking University.

## Author contributions

X. Lei and Y. Li conceived the project. J. Bai performed the organic synthesis, protein modification, cell labelling and amplification, confocal imaging and fluorescence quantification. F. Guo performed the mouse treatment, the surgery and mouse intestinal frozen section preparation. M. Li participated in the early-stage setup and experimental design. All authors contributed to the data processing and analysis. J. Bai and X. Lei wrote the manuscript with input from the other authors.

## Conflicts of interest

There are no conflicts to declare.

## Supplementary Material

CB-002-D1CB00002K-s001
