# Supplementary material for: Click-based amplification: designed to facilitate various target labelling modes with ultralow background amplification
Source: RSC Chem Biol. 2021 Mar 20;2(3):906–16. doi: 10.1039/d1cb00002k (PMC8341700; doi:10.1039/d1cb00002k)
Supplement: CB-002-D1CB00002K-s001 [file CB-002-D1CB00002K-s001.pdf]

Electronic Supplementary Material (ESI) for RSC Chemical Biology.
This journal is © The Royal Society of Chemistry 2021

Click-based amplification: designed to facilitate various target labelling with ultralow background

Jinyi Bai, Fusheng Guo, Mengyao Li, Yulong Li * and Xiaoguang Lei *

Supplementary Information

Table of Contents

Supplementary Information....1
Fig. S1 CuAAC-type converter showed ultralow nonspecific amplification....2
Fig. S2 Quantification of the amount of modifications on streptavidin. 3
Fig. S3 Optimization of Click-based amplification conditions in context of fixed HeLa cell....4
Fig. S4 An instance of how cells were chosen randomly for fluorescence quantification....5
Fig. S5 A repeated amplification experiment of NHS-N_3 labelling in HeLa cells....6
Fig. S6 Optimization of the click conversion of EdU labelling in HeLa cells....7
Fig. S7 Optimization of the concentration of SA-HRP for TSA Amplification....8
Table S1 Raw data for Figrue 4E with Click-based Amplification....8
Fig. S8 A repeated amplification experiment of Afatinib-N_3 labelling in HeLa cells. 9
Fig. S9 Synthesis of NHS-N_3 (2) 10
Fig. S10 Synthesis of NHS-pAz (6) 10
Fig. S11 Synthesis of Biotin-PEG_2-DIBO (10) 11
Fig. S12 Synthesis of Biotin-N_3 (13)....12
Fig. S13 ^{1}H NMR spectrum of NHS-N_3 (2) 13
Fig. S14 ^{13}C NMR spectrum of NHS-N_3 (2) 14
Fig. S15 ^{1}H NMR spectrum of NHS-pAz (6) 15
Fig. S16 ^{1}H NMR spectrum of Biotin-PEG_2-DIBO (10) 16
Fig. S17 ^{1}H NMR spectrum of Biotin-N_3 (13)....17
Fig. S18 ^{13}C NMR spectrum of Biotin-N_3 (13)....18
Supplementary References....18

Fig. S1 CuAAC-type converter showed ultralow nonspecific amplification.

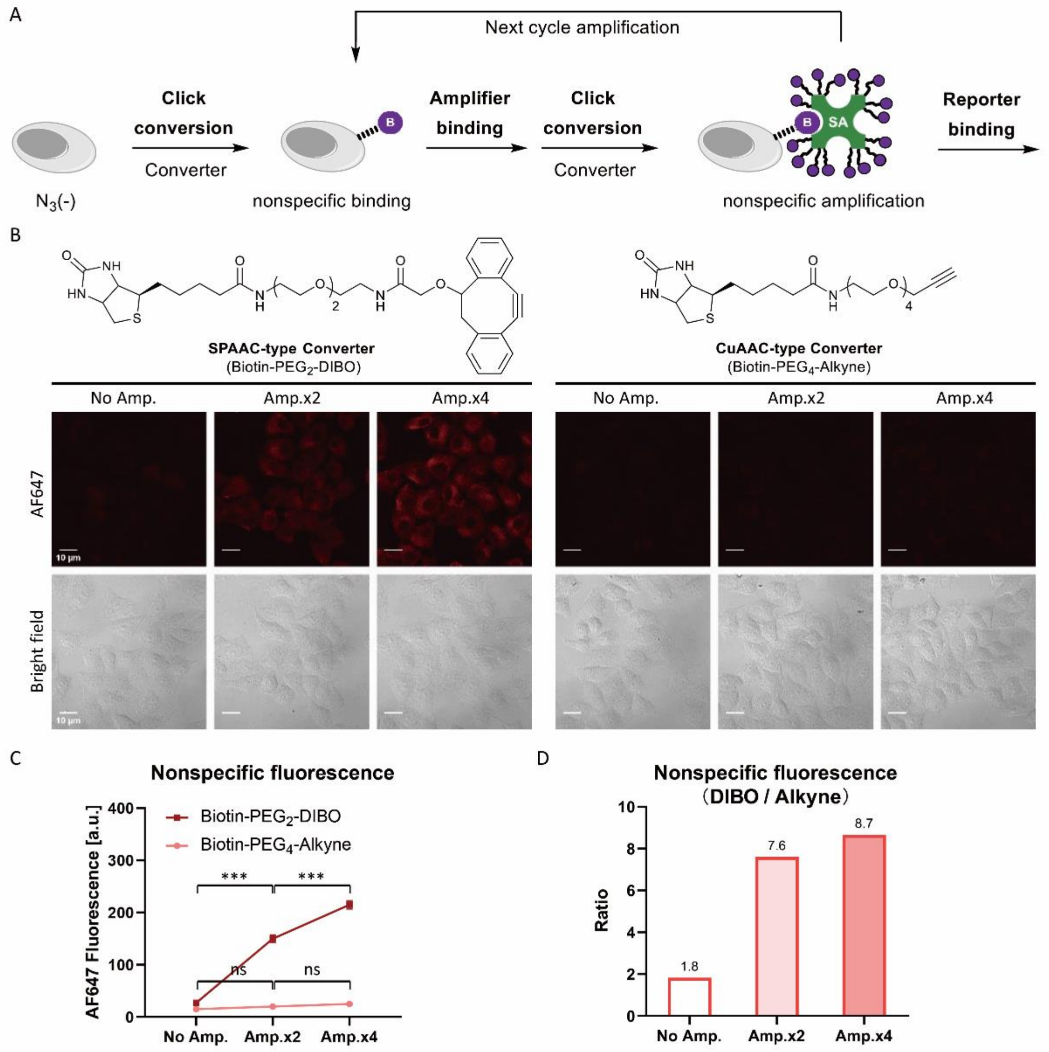

Fig. S1 CuAAC-type converter showed ultralow nonspecific amplification. (A) Scheme of nonspecific amplification evaluation of Click-based amplification. Amplification was performed on fixed cell without any click-labelling. (B) SPAAC-type converter Biotin-PEG$_2$-DIBO introduced much stronger nonspecific fluorescence than CuAAC-type converter Biotin-PEG$_4$-Alkyne. Amp.x2 and Amp.x4 represent Click-based amplifications were carried out twice and four times before reporter binding, respectively. (C) Fluorescence quantification of confocal imaging in (B) with ImageJ. Dark red line represents the cellular fluorescence intensity using Biotin-PEG$_2$-DIBO, and each data point is the average fluorescence intensity of 16 cells chosen randomly from the microscope imaging. Light red line represents the cellular fluorescence intensity using Biotin-PEG$_4$-Alkyne, and each data point is the average fluorescence intensity of 16 cells chosen randomly from the microscope imaging. Error bar: the standard error (SE). (D) Ratios of nonspecific fluorescence with Biotin-PEG$_2$-DIBO to Biotin-PEG$_4$-Alkyne. Scale bar, 10 µm. *** = $p < 0.001$, ns = no significant.

Fig. S2 Quantification of the amount of modifications on streptavidin.

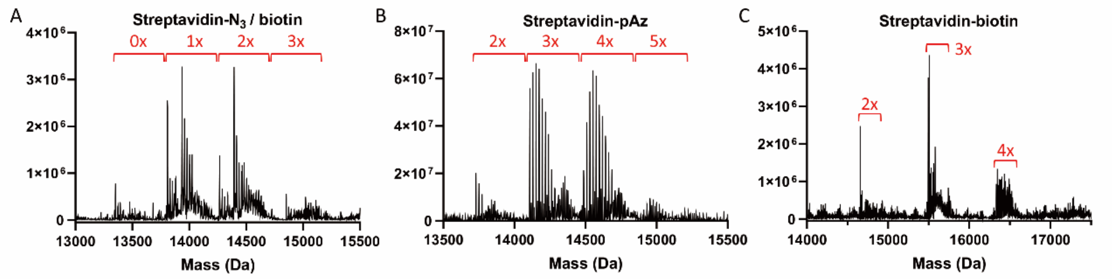

Number of biotin	0x	1x	2x	3x
Intergration (%)	7.2	43.1	39.6	10.2

Average number of biotin: 1.53

Number of pAz	2x	3x	4x	5x
Intergration (%)	12.5	37.2	39.6	10.7

Average number of pAz: 3.48

Number of biotin	2x	3x	4x
Intergration (%)	18.7	47.1	34.3

Average number of biotin: 3.16

Fig. S2 Quantification of the amount of modifications on streptavidin. (A) Up: the deconvoluted ESI-TOF mass spectra of Streptavidin-N_3 / biotin (Figure 2E); down: the integral area (%) of MS peaks and the calculated number of biotin tags. (B) Up: the deconvoluted ESI-TOF mass spectra of Streptavidin-pAz (Figure 2F); down: the integral area (%) of MS peaks and the calculated number of pAz tags. (C) Up: the deconvoluted ESI-TOF mass spectra of Streptavidin-biotin (Figure 2G); down: the integral area (%) of MS peaks and the calculated number of biotin tags.

Fig. S3 Optimization of Click-based amplification conditions in context of fixed HeLa cell.

To obtain better amplification efficiency in cell imaging, we optimized the reaction conditions in the context of fixed HeLa cells, which were labelled with 1 μM of NHS-N_3 on ice for 30 min and fixed with PFA (Fig. S3A). The results showed that in amplifier binding step, 0.1-0.5 μg•mL-1 of streptavidin-pAz obtained best amplification efficiency and signal-to-noise ratio (Fig. S3B), and BSA blocking had no significant improvement for amplification (Fig. S3C). In click conversion step, the optimized concentrations of biotin-PEG_4-alkyne and copper catalyst were 10 μM and 100 μM, respectively (Fig. S3D and S3E).

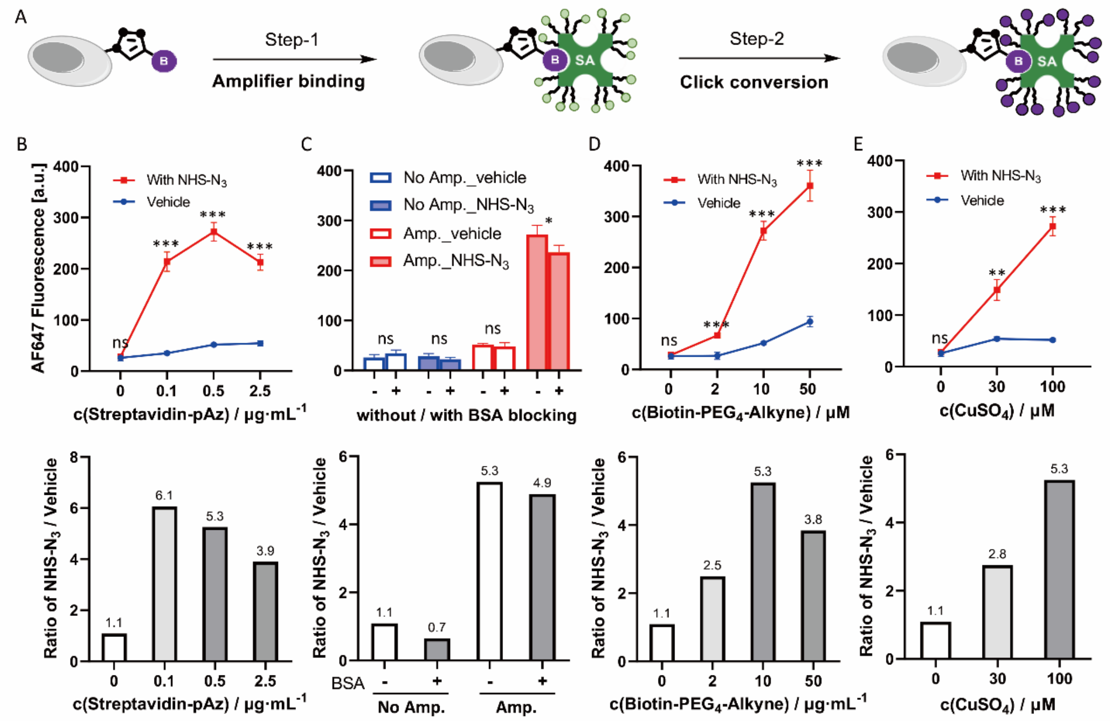

Fig. S3 Optimization of Click-based amplification conditions in context of fixed HeLa cell. (A) Scheme of Click-based amplification. HeLa cell was treated with 1 μM of NHS-N_3 on ice for 30 min, and then fixed with 4% PFA. Click conversion of labelled N_3 to biotin was performed before Click-based amplification. (B) Amplifier binding step with a concentration gradient of streptavidin-pAz from 0.1 μg/mL to 2.5 μg/mL. Up, quantification of cellular AF647 fluorescence intensity of fixed HeLa cells with and without NHS-N_3 treatment, and each data point is the average fluorescence intensity of 20 cells chosen randomly from the microscope imaging. Error bar: the standard error (SE). Down, ratios of cellular AF647 fluorescence with NHS-N_3 treatment to vehicle control. (C) Amplifier binding were performed with and without BSA blocking. (D) Click conversion step with a concentration gradient of Biotin-PEG_4-Alkyne from 2 μM to 50 μM. (E) Click conversion step with a concentration gradient of $CuSO_4$ catalyst from 30 μM to 100 μM, and 200 μM of BTTAA was added as the ligand of cupper. (B)-(E) share the same fluorescence quantification methods. *** = $p < 0.001$, ** = $p < 0.01$, ns = no significant.

Fig. S4 An instance of how cells were chosen randomly for fluorescence quantification.

Take the microscope imaging in Figure 3A (1 μM of NHS-N_3, Amp.) as an example. Two microscope images were acquired for one testing sample, and one image (Field 2) was present in Figure 3A. Ten cells and one background field were chosen randomly from each image. First, open nd2 files with ImageJ and switch to AF647 channel (Fig. S4A). The image contrast was adjusted to show the edges of HeLa cells clearly using Image -> Adjust -> Brightness/Contrast -> Set (Fig. S4B). Circle one cell along its edge using Freehand selections, then quantify the fluorescence intensity using Analyze -> Measure, and write down the Mean value. Repeat this step to quantify the fluorescence intensity of 20 cells and 2 background fields (Fig. S4C). To minimize the artificial deviation, one can select cells in bright field channel first, and then quantify the fluorescence intensity of selected cells in AF647 channel.

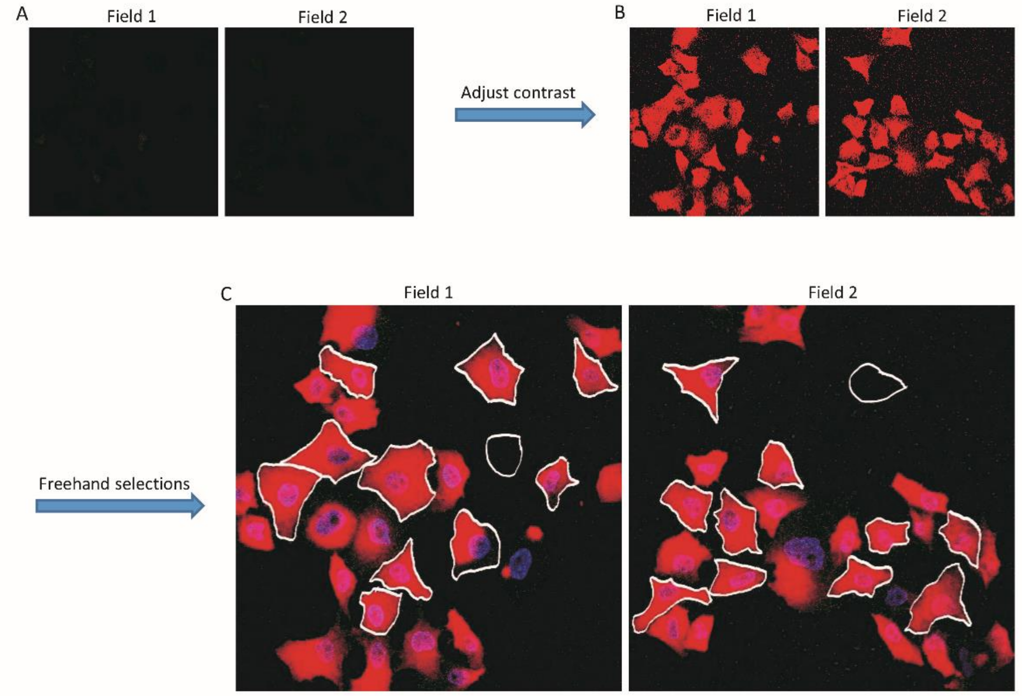

Fig. S4 An instance of how cells were chosen randomly for fluorescence quantification. (A) Open nd2 files with ImageJ and switch to AF647 channel. (B) The image contrast was adjusted to show the edges of HeLa cells clearly using Image -> Adjust -> Brightness/Contrast -> Set. (C) Circle one cell along its edge using Freehand selections, then quantify the fluorescence intensity using Analyze -> Measure, and write down the Mean value. Repeat this step to quantify the fluorescence intensity of 20 cells and 2 background fields.

Fig. S5 A repeated amplification experiment of NHS-N_3 labelling in HeLa cells.

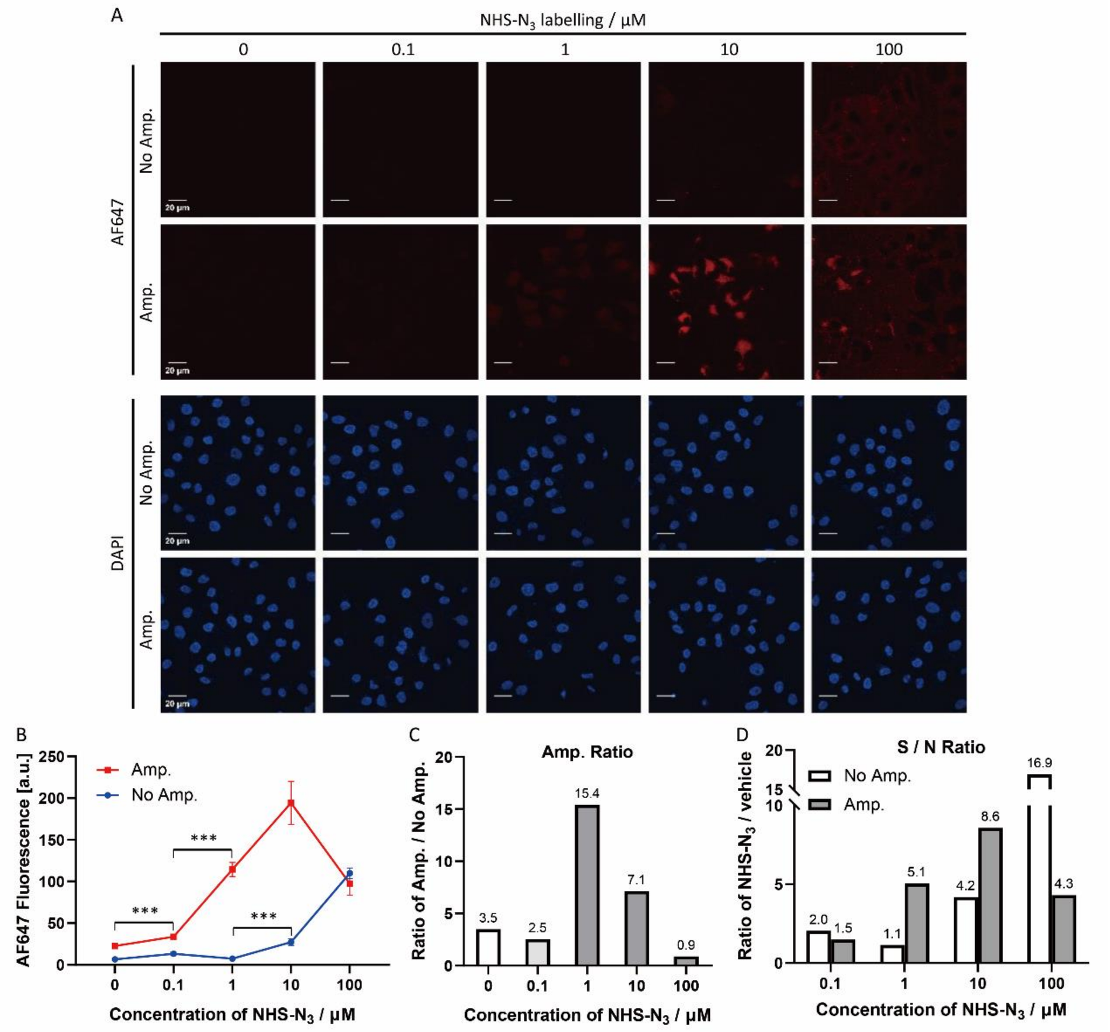

Fig. S5 A repeated amplification experiment of NHS-N_3 labelling in HeLa cells. (A) Confocal imaging of fixed HeLa cells without and with Click-based amplification (No Amp. and Amp.). HeLa cells were treated with a concentration gradient of NHS-N_3 from 0.1 µM to 100 µM. DAPI indicated the nucleus of the HeLa cell. Scale bar, 20 µm. (B) Quantification of cellular AF647 fluorescence intensity in (A) with ImageJ. Red line represents the fluorescence intensity of HeLa cells with Click-based amplification (Amp.), and each data point is the average fluorescence intensity of 20 cells chosen randomly from the microscope imaging. Blue line, the fluorescence intensity of HeLa cells without Click-based amplification (No Amp.). Error bar: the standard error (SE). *** = $p < 0.001$. (C) Amplification ratios (Amp. / No Amp.). (D) Signal-to-noise ratios (NHS-N_3 / vehicle).

Fig. S6 Optimization of the click conversion of EdU labelling in HeLa cells.

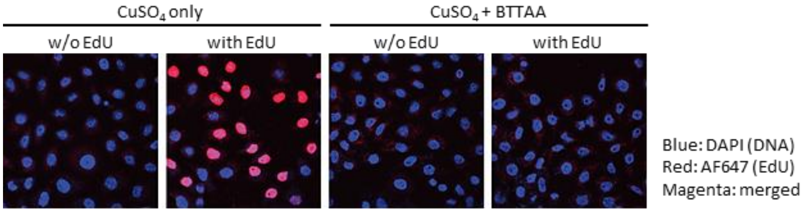

Fig. S6 Optimization of the click conversion of EdU labelling in HeLa cells. Merged confocal imaging of fixed HeLa cells with EdU labelling: blue channel indicated the nucleus of the HeLa cell stained with DAPI, red channel indicated the signal of EdU labelling after click reaction with biotin-N_3 and coupling with streptavidin-AF647, and magenta channel indicated the colocalization of EdU labelling with DNA. Click condition: 10 µM of biotin-N_3, 100 µM of $CuSO_4$, and 2.5 mM of sodium ascorbate in PBS, with or without 200 µM of BTTAA. The strong AF647 fluorescence signal showed click conversion of EdU occurred with $CuSO_4$ only, while BTTAA slowed down the click conversion of EdU in HeLa cells.

Fig. S7 Optimization of the concentration of SA-HRP for TSA Amplification.

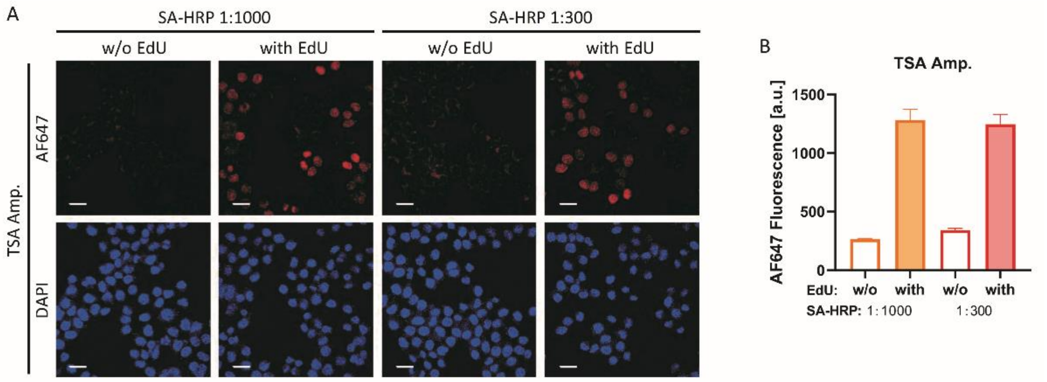

Fig. S7 Optimization of the concentration of SA-HRP for TSA Amplification. (A) Confocal imaging of fixed HeLa cells with TSA amplification. The concentrations of SA-HRP used in TSA were 1:1000 and 1:300. HeLa cells were treated with 10 μM of EdU in DMEM at 37 ℃ for overnight. DAPI indicated the nucleus of the HeLa cell. (B) Quantification of cell fluorescence intensity in (A). For w/o EdU groups, all the cell nucleuses were dark, and 20 cells was chosen randomly to average the fluorescence intensity. For with EdU groups, all the lighted nucleuses were measured to average the fluorescence intensity. Scale bar, 20 μm.

Table S1 Raw data for Figrue 4E with Click-based Amplification.

1. No Amp., w/o EdU. 20 cells were chosen randomly

131.363	130.946	128.574	128.685	127.911	128.698	129.100	129.268	128.685	127.652
127.008	127.838	128.464	129.071	128.562	127.641	127.833	126.917	130.144	127.170
**Background fluorescence:**			122.782	122.782					
**Average cell fluorescence:**			5.776		**SE:**	0.268			

2. No Amp., with EdU. All the 30 lighted nucleuses were measured

927.431	838.826	515.415	510.300	608.226	488.072	483.097	598.771	217.883	812.801
178.631	244.523	454.518	534.803	375.194	370.170	518.715	200.634	819.384	664.945
707.290	423.331	446.265	360.919	515.551	164.510	394.086	385.945	182.400	173.602
**Background fluorescence:**			122.937	122.884					
**Average nucleus fluorescence:**				347.631	**SE:**	38.805			

3. Click-based Amp., w/o EdU. 20 cells were chosen randomly

160.393	157.850	158.752	156.972	163.897	153.454	158.268	154.688	152.930	151.186
149.104	149.184	147.035	146.613	146.218	149.853	145.443	152.876	156.519	149.396
**Background fluorescence:**			123.942	124.038					
**Average cell fluorescence:**			29.042		**SE:**	5.028			

4. Click-based Amp., with EdU. All the 21 lighted nucleuses were measured

1765.350	1704.599	1219.683	1762.522	1618.057	1890.259	1923.729	472.715	1380.003	1279.158
1139.309	1092.736	1162.938	1439.554	549.516	777.380	579.032	433.045	927.051	980.657
567.945									
**Background fluorescence:**			123.413	123.170					
**Average nucleus fluorescence:**				1051.244	**SE:**	106.812			

Fig. S8 A repeated amplification experiment of Afatinib-N_3 labelling in HeLa cells.

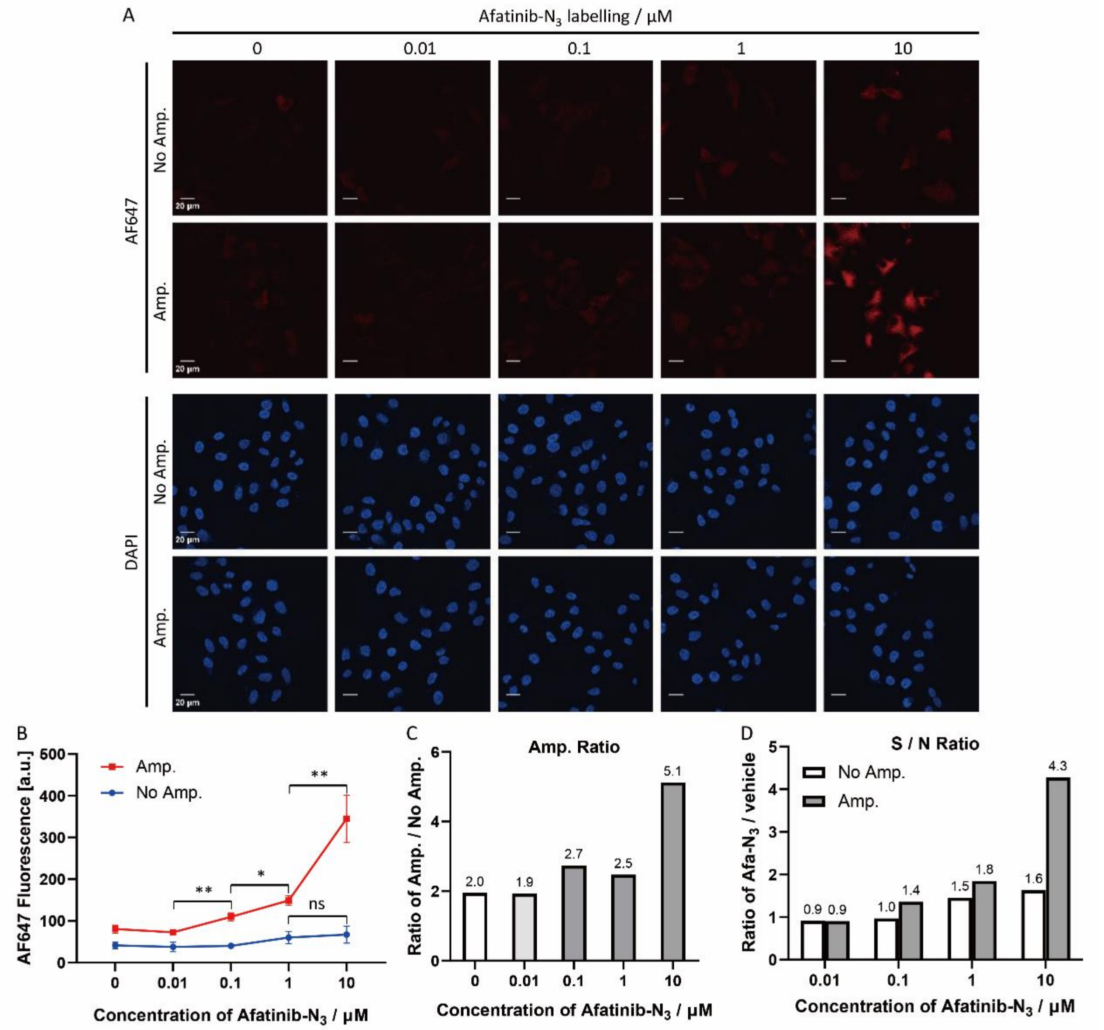

Fig. S8 A repeated amplification experiment of Afatinib-N_3 labelling in HeLa cells. (A) Confocal imaging of fixed HeLa cells without and with Click-based amplification (No Amp. and Amp.). HeLa cells were treated with a concentration gradient of Afatinib-N_3 from 0.01 μM to 10 μM in DMEM at 37 ℃ for 1 h. DAPI indicated the nucleus of the HeLa cell. Scale bar, 20 μm. (B) Quantification of cellular AF647 fluorescence intensity in (A) with ImageJ. Red line represents the fluorescence intensity of HeLa cells with Click-based amplification (Amp.), and each data point is the average fluorescence intensity of 20 cells chosen randomly from the microscope imaging. Blue line, the fluorescence intensity of HeLa cells without Click-based amplification (No Amp.). Error bar: the standard error (SE). ** = $p < 0.005$, * = $p < 0.05$, ns = no significant. (C) Amplification ratios (Amp. / No Amp.). (D) Signal-to-noise ratios (Afatinib-N_3 / vehicle).

Fig. S9 Synthesis of NHS-N$_3$ (2)

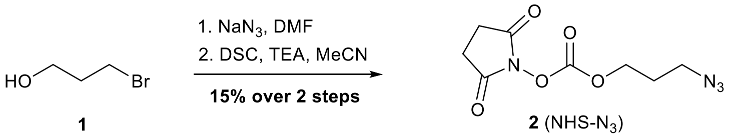

Fig. S9 Synthesis of NHS-N$_3$ **2**.

3-azidopropyl (2,5-dioxopyrrolidin-1-yl) carbonate (2) was synthesized following the methodology developed by F.C. Wu et al.[1] 3-Bromopropan-1-ol **1** (226 µL, 2.5 mmol) was added to a stirred solution of NaN_3 (230 mg, 3.75 mmol) in dry DMF (5 mL). The reaction mixture was stirred at room temperature for 1 d, and then 25 mL of Et_2O/*n*-hexane (1:1) was added. The organic phase was washed with brine, and dried over anhydrous Na_2SO_4. After filtration, the filtrate was concentrated *in vacuo*. The crude intermediate 3-azidopropan-1-ol was used in next step without further purification. *N, N'*-disuccinimidyl carbonate (DSC, 0.96 g, 3.75 mmol) was added in one portion to a solution of the crude 3-azidopropan-1-ol (≤ 2.5 mmol) in dry MeCN (10 mL), then triethylamine (TEA, 1.04 mL, 7.5 mmol) was added dropwise. The reaction mixture was stirred at room temperature for 40 min. The solvent was removed *in vacuo*, and the residue was redissolved in ethyl acetate (50 mL). The organic phase was washed with sat. $NaHCO_3$ and brine, and dried over anhydrous Na_2SO_4. After filtration, the filtrate was concentrated *in vacuo*. The residue was purified by column chromatography with silica gel (20%-50% ethyl acetate in petroleum ether) to afford compound **2** (92.8 mg, 15% over two steps) as a colorless oil; ^{1}H NMR (400 MHz, $CDCl_3$) δ(ppm) 4.42 (t, 2H, *J* = 6.1 Hz), 3.47 (t, 2H, *J* = 6.6 Hz), 2.84 (s, 4H), 2.01 (m, 2H); ^{13}C NMR (101 MHz, $CDCl_3$) δ(ppm) 168.6, 151.5, 68.1, 47.5, 28.0, 25.5; HRMS: $[M+H]^+$ calculated for $C_8H_{11}N_4O_5$ 243.0724, found 243.0720.

Fig. S10 Synthesis of NHS-pAz (6)

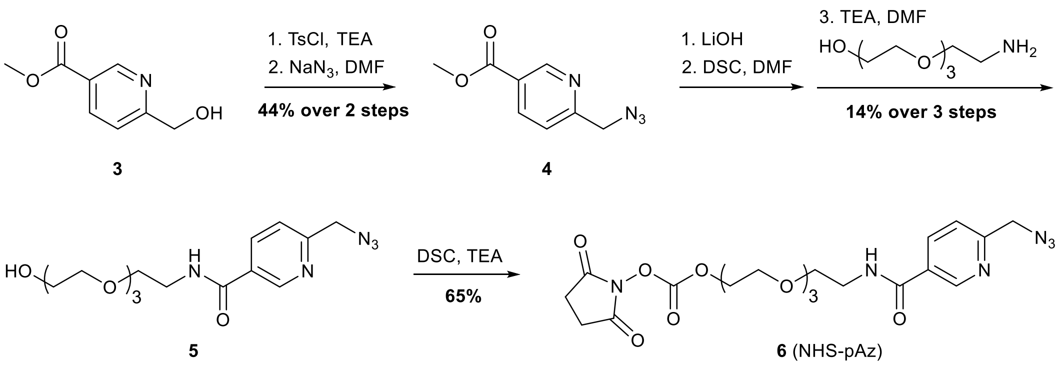

Fig. S10 Synthesis of NHS-pAz **6**.

Methyl 5-(azidomethyl)nicotinate (4) was synthesized following the methodology developed by Uttamapinant et al.[2] Para-toluenesulfonyl chloride (TsCl, 2.74 g, 14.4 mmol) and TEA (6.65 mL, 47.9 mmol) was added slowly to a solution of methyl 6-(hydroxymethyl)nicotinate **3** (1.60 g, 9.6 mmol) in dry CH_2Cl_2 (100 mL). The reaction was stirred at room temperature for 3 h, and then CH_2Cl_2 was removed *in vacuo*. The residue was redissolved in dry THF (80 mL), and NaN_3 (6.22 g, 96 mmol) was added. The reaction mixture was further stirred at room temperature for 40 h, monitored by LC-MS for a complete conversion. Most solvent was removed *in vacuo*. The residue was redissolved in 100 mL of ethyl acetate/water (1:1). The aqueous layer was further extracted with ethyl acetate three times. The combined organic phase was washed with brine, and dried over anhydrous Na_2SO_4. After filtration, the filtrate was concentrated *in vacuo*. The residue was purified by column chromatography with silica gel (5%-15% ethyl acetate in petroleum ether) to afford compound **4** (806.7 mg, 44% over two steps) as a light yellow solid; ^{1}H NMR (400 MHz, $CDCl_3$) δ(ppm) 9.19 (d, 1H, *J* = 2.0 Hz), 8.33 (dd, 1H, *J* = 8.5, 2.0 Hz), 7.45 (d, 1H, *J* = 8.5 Hz), 4.58 (s, 2H), 3.96 (s, 3H); ^{13}C NMR (101 MHz, $CDCl_3$) δ(ppm) 165.5, 160.1, 150.8, 138.2, 125.3, 121.3, 55.4, 52.5; HRMS: $[M+H]^+$ calculated for $C_8H_9N_4O_2$ 193.0720, found 193.0720.

6-(azidomethyl)-N-(2-(2-(2-(2-hydroxyethoxy)ethoxy)ethoxy)ethyl)nicotinamide (5) To a solution of **4** (114.0 mg, 0.59 mmol) in

methanol (2.5 mL), was added aqueous solution of LiOH (1.0 M, 1.78 mL). The reaction mixture was stirred at room temperature for 30 min, and then quenched by 60 µL of acetic acid (1.05 mmol). The mixture was loaded directly onto a silica gel column equilibrated with ethyl acetate + 1% acetic acid. The intermediate was washed out with ethyl acetate + 1% acetic acid + 4% acetonitrile, and concentrated *in vacuo* to afford a yellow solid. The hydrolyzed intermediate was used in next step without further purification. *N, N'*-disuccinimidyl carbonate (DSC, 302.3 mg, 1.18 mmol) was added in one portion to a solution of the hydrolyzed intermediate (≤ 0.59 mmol) in dry DMF (1.5 mL), then TEA (421.4 µL, 3.0 mmol) was added dropwise. The reaction mixture was stirred at room temperature for 3 h. The mixture was diluted with 50 mL of $CHCl_3/H_2O$ (1:1). The aqueous layer was further extracted with $CHCl_3$ three times. The combined organic phase was washed with brine, dried over anhydrous Na_2SO_4, and concentrated *in vacuo*. The ester intermediate was purified by flash column chromatography with silica gel (40% ethyl acetate in petroleum ether) to afford a clear liquid containing DMF. A solution of 2-(2-(2-(2-aminoethoxy)ethoxy)ethoxy)ethan-1-ol (228.0 mg, 1.18 mmol) in DMF (0.5 mL) was added dropwise to the ester intermediate, then TEA (253 µL, 1.78 mmol) was added dropwise. The reaction mixture was stirred at room temperature overnight, and then the solvent was removed *in vacuo*, and the resulting residue was purified by column chromatography with silica gel (1%-5% MeOH in CH_2Cl_2) to afford compound **5** (28.2 mg, 14% over three steps) as a light yellow oil; ^{1}H NMR (400 MHz, $CDCl_3$) δ(ppm): 9.06 (d, 1H, *J* = 2.0 Hz), 8.25 (dd, 1H, *J* = 8.5, 2.0 Hz), 8.16 (s, 1H), 7.42 (d, 1H, *J* = 8.5 Hz), 4.53 (s, 2H), 3.78-3.57 (m, 14H), 3.55-3.42 (m, 2H); ESI-MS: $[M+H]^+$ $C_{15}H_{24}N_5O_5$ 354.6.

1-(6-(azidomethyl)pyridin-3-yl)-1-oxo-5,8,11-trioxa-2-azatridecan-13-yl (2,5-dioxopyrrolidin-1-yl) carbonate (6) *N, N'*-disuccinimidyl carbonate (DSC, 69.5 mg, 0.27 mmol) was added in one portion to a solution of **5** (27.3 mg, 0.077 mmol) in dry MeCN (1.5 mL), then TEA (75 µL, 0.54 mmol) was added dropwise. The reaction mixture was stirred at room temperature for 2.5 h. The solvent was removed *in vacuo*, and the residue was purified by column chromatography with silica gel (1%-6% MeOH in CH_2Cl_2) to afford compound **6** (24.7 mg, 65%) as a thick oil; ^{1}H NMR (400 MHz, $CDCl_3$) δ(ppm): 9.00 (d, 1H, *J* = 2.0 Hz), 8.19 (dd, 1H, *J* = 2.3 Hz, 8.1 Hz), 7.43 (d, 1H, *J* = 8.1 Hz), 7.09 (s, 1H), 4.54 (s, 2H), 4.44 (m, 2H), 3.75 (m, 2H), 3.71-3.64 (m, 12H), 2.83 (s, 4H); HRMS: $[M+H]^+$ calculated for $C_{20}H_{27}N_6O_9$ 495.1834, found 495.1829.

Fig. S11 Synthesis of Biotin-PEG$_2$-DIBO (10)

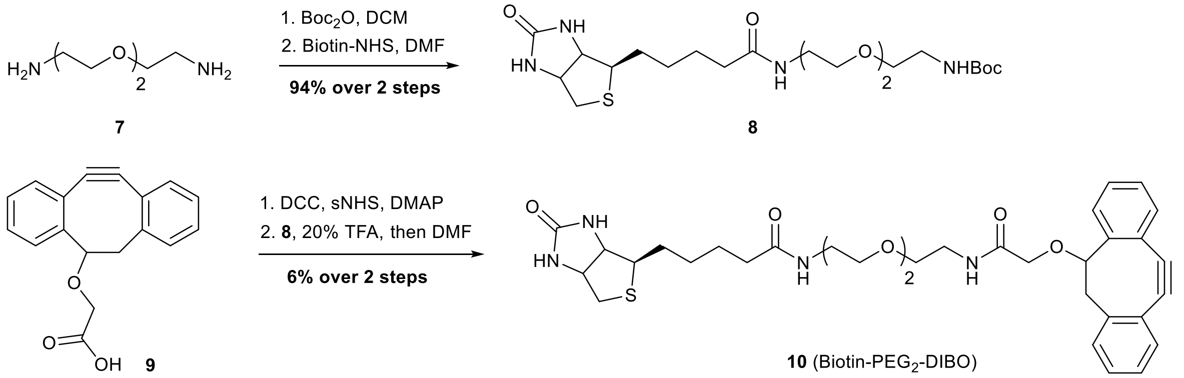

Fig. S11 Synthesis of Biotin-PEG$_2$-DIBO **10**.

Biotin-PEG$_2$-NHBoc (8) To an ice-cooled solution of 2,2'-(ethane-1,2-diylbis(oxy))bis(ethan-1-amine) **7** (438 µL, 3.0 mmol) in CH_2Cl_2 (30 mL) was added a solution of di-tert-butyldicarbonate (Boc_2O, 103 µL, 0.45 mmol) in CH_2Cl_2 (2 mL) under vigorous stirring. The reaction mixture was further stirred in ice bath for 5 h, and then warmed to room temperature overnight. The mixture was washed with brine twice, dried over anhydrous Na_2SO_4, and concentrated *in vacuo* to afford the crude mono-Boc intermediate. To a solution of the intermediate (≤ 0.45 mmol) in dry DMF (4 mL) was added biotin-NHS powder (102.4 mg, 0.30 mmol) in one portion, then TEA (207 µL, 1.5 mmol) was added dropwise. The reaction mixture was stirred at room temperature overnight. The solvent was removed *in vacuo*, and the residue was purified by column chromatography with silica gel (5%-10% MeOH in $CHCl_3$) to afford compound **8** (134.3 mg, 94%) as a white solid; ^{1}H NMR (400 MHz, $CDCl_3$) δ(ppm): 6.45 (s, 1H), 6.03 (s, 1H), 5.17 (s, 1H), 5.10 (s, 1H), 4.55-4.47 (m, 1H), 4.36-4.29 (m,

1H), 3.72 (q, 2H, *J* = 7.0 Hz), 3.67-3.53 (m, 8H), 3.48-3.42 (m, 2H), 3.16 (q, 1H, *J* = 4.6 Hz), 2.96-2.88 (m, 1H), 2.74 (d, 1H, *J* = 12.8 Hz), 2.24 (t, 2H, *J* = 7.4 Hz), 1.80-1.60 (m, 4H), 1.50-1.40 (s, 11H); ESI-MS: $[M+H]^+$ $C_{21}H_{39}N_4O_6S$ 475.4.

Biotin-PEG$_2$-DIBO (10) Dibenzocyclooctyne acid **9** (DIBO-COOH, 55.7 mg, 0.2 mmol), N-hydroxysulfosuccinimide sodium salt (sNHS, 43.4 mg, 0.2 mmol) and 4-dimethylaminopyridine (DMAP, 1.2 mg, 0.01 mmol) were dissolved in DMF (0.5 mL), to which a solution of dicyclohexylcarbodiimide (DCC, 45.4 mg, 0.22 mmol) in DMF (0.75 mL) was added. The reaction mixture was stirred at room temperature overnight, and then cooled in ice bath for 2 h. The generated solid was removed by filtration, and the filtrate was added Et_2O (10 mL) under vigorous stirring. The newly generated solid was collected by filtration, washed with Et_2O, and concentrated *in vacuo* to afford the DIBO intermediate as a white solid (51.2 mg, ≤ 54%). **8** (99.7 mg, 0.21 mmol) was dissolved in 1 mL of TFA/CH_2Cl_2 (1:4), and stirred for 1.5 h. The mixture was concentrated *in vacuo*, and redissolved in dry DMF (1 mL). A solution of the DIBO intermediate (48.0 mg, ≤ 0.1 mmol) and TEA (138 µL, 1.0 mmol) in dry DMF (1 mL) was added to the reaction mixture. The mixture was stirred at room temperature overnight, and then the solvent was removed *in vacuo*, and the residue was purified by column chromatography with silica gel (1%-10% MeOH in CH_2Cl_2) to afford compound **10** (7.6 mg, 6% over 2 steps) as a white solid; ^{1}H NMR (400 MHz, MeOH-d_4) δ(ppm): 7.69 (d, 1H, *J* = 7.8 Hz), 7.55 (d, 1H, *J* = 7.2 Hz), 7.47-7.41 (m, 1H), 7.39-7.30 (m, 5H), 4.49-4.43 (m, 1H), 4.29-4.21 (m, 2H), 4.06 (s, 2H), 3.61 (s, 4H), 3.53 (m, 4H), 3.43 (m, 2H), 3.37-3.33 (m, 3H), 3.19-3.10 (m, 1H), 2.93-2.85 (m, 1H), 2.81-2.73 (m, 1H), 2.68 (d, 1H, *J* = 12.7 Hz), 2.18 (t, 2H, *J* = 7.4 Hz), 1.76-1.50 (m, 4H), 1.40 (q, 2H, *J* = 7.6 Hz); HRMS: $[M+H]^+$ calculated for $C_{34}H_{43}N_4O_6S$ 635.2898, found 635.2900.

Fig. S12 Synthesis of Biotin-N$_3$ (13)

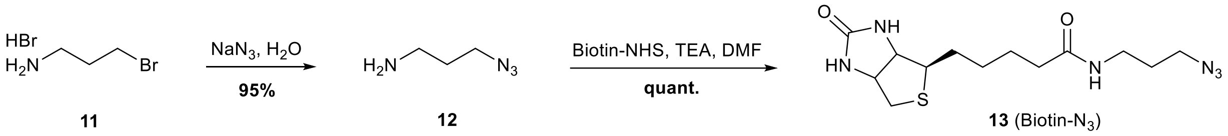

Fig. S12 Synthesis of Biotin-N$_3$ **13**.

3-azidopropan-1-amine (12) To an aqueous solution of 3-bromopropan-1-amine hydrobromide **11** (3.28 g, 1.5 M), was added an aqueous solution of NaN_3 (3.25 g, 3 M). The reaction mixture was heated to reflux and stirred for 16 h, then cooled in the ice bath. KOH pellets (4 g, 71 mmol) was added to the mixture under vigorous stirring, and the mixture was extracted with Et_2O three times. The combined organic phase was dried over anhydrous Na_2SO_4, and concentrated *in vacuo* to afford compound **12** (1.42 g, 95%) as a light yellow oil; ^{1}H NMR (400 MHz, $CDCl_3$) δ(ppm): 3.37 (t, 2H, *J* = 6.7Hz), 2.80 (t, 2H, *J* = 6.8Hz), 1.73 (m, 2H), 1.5-1.0 (s, 2H); ^{13}C NMR (101 MHz, $CDCl_3$) δ(ppm) 49.2, 39.4, 32.5.

Biotin-N$_3$ (13) To a solution of biotin-NHS (102.4 mg, 0.30 mmol) in dry DMF (1 mL), was added **12** (89 µL, 0.90 mmol) and TEA (208 µL, 1.50 mmol). The reaction mixture was stirred at room temperature overnight. The solvent was removed *in vacuo*, and the residue was purified by column chromatography with silica gel (3%-15% MeOH in $CHCl_3$) to afford compound **13** (98.0 mg, quant.) as a white solid; ^{1}H NMR (400 MHz, MeOH-d_4): 4.52-4.45 (m, 1H), 4.33-4.26 (m, 1H), 3.35 (t, 2H, *J* = 6.7 Hz), 3.24 (t, 2H, *J* = 6.7 Hz), 3.24-3.16 (m, 1H), 2.96-2.88 (m, 1H), 2.70 (d, 1H, *J* = 12.7 Hz), 2.20 (t, 2H, *J* = 7.4 Hz), 1.80-1.53 (m, 6H), 1.44 (q, 2H, *J* = 7.7 Hz); ^{13}C NMR (101 MHz, MeOH-d_4) δ(ppm) 174.8, 164.8, 62.0, 60.2, 55.6, 48.7, 39.6, 36.3, 35.4, 28.4, 28.4, 28.1, 25.5, 24.9; HRMS: $[M+H]^+$ calculated for $C_{13}H_{23}N_6O_2S$ 327.1598, found 327.1592.

Fig. S13 ^{1}H NMR spectrum of NHS-N_3 (2)

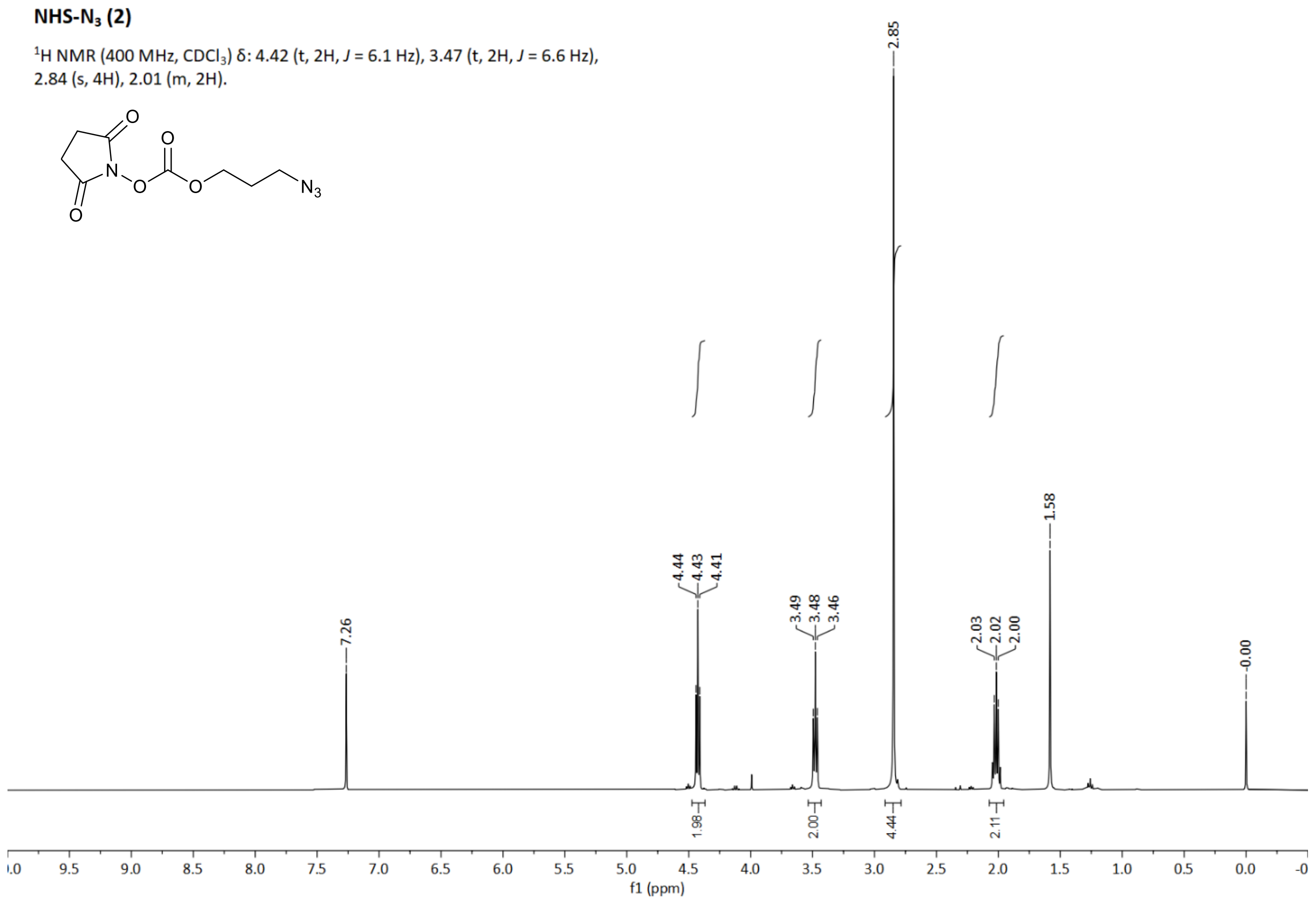

Fig. S13 ^{1}H NMR spectrum of NHS-N_3 **2**

Fig. S14 ^{13}C NMR spectrum of NHS-N_3 (2)

NHS-N_3 (2)

^{13}C NMR (101 MHz, $CDCl_3$) δ 168.60, 151.50, 68.10, 47.45, 27.99, 25.47.

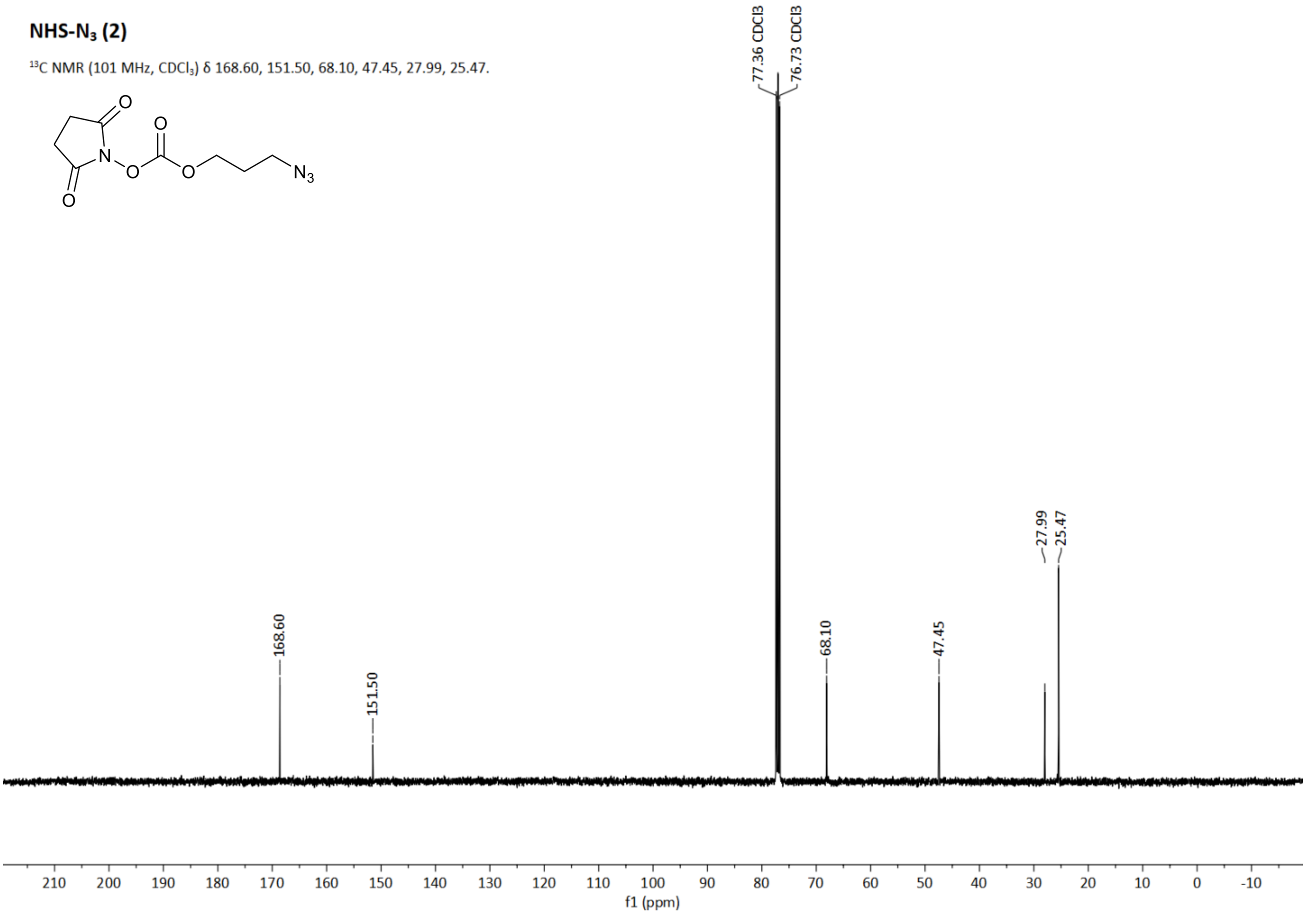

Fig. S14 ^{13}C NMR spectrum of NHS-N_3 **2**

Fig. S15 ^{1}H NMR spectrum of NHS-pAz (6)

NHS-pAz (6)

^{1}H NMR (400 MHz, $CDCl_3$) δ: 9.00 (d, 1H, *J* = 2.0 Hz), 8.19 (dd, 1H, *J* = 2.3 Hz, 8.1 Hz), 7.43 (d, 1H, *J* = 8.1 Hz), 7.09 (s, 1H), 4.54 (s, 2H), 4.44 (m, 2H), 3.75 (m, 2H), 3.71-3.64 (m, 12H), 2.83 (s, 4H).

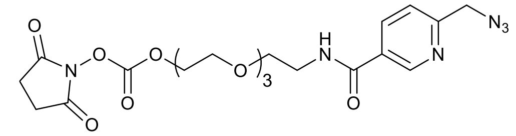

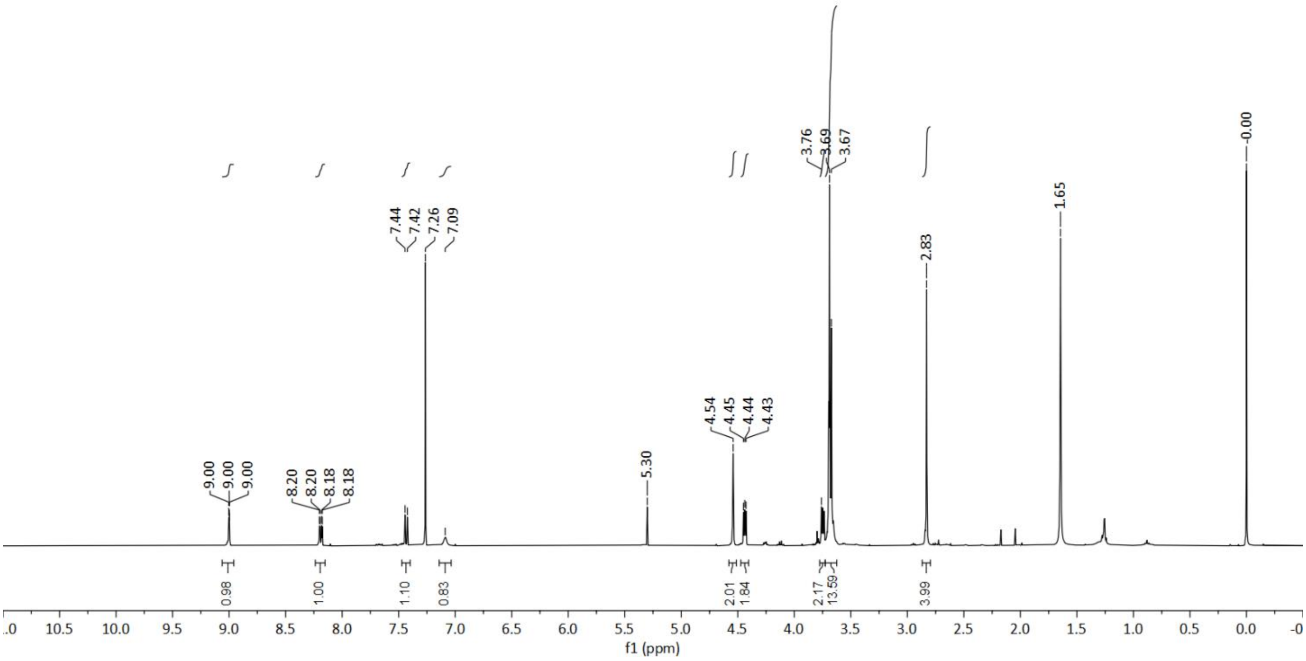

Fig. S15 ^{1}H NMR spectrum of NHS-pAz **6**

Fig. S16 ^{1}H NMR spectrum of Biotin-PEG$_2$-DIBO (10)

Biotin-PEG$_2$-DIBO (10)

^{1}H NMR (400 MHz, MeOH-d4) δ: 7.69 (d, 1H, J = 7.8 Hz), 7.55 (d, 1H, J = 7.2 Hz), 7.47-7.41 (m, 1H), 7.39-7.30 (m, 5H), 4.49-4.43 (m, 1H), 4.29-4.21 (m, 2H), 4.06 (s, 2H), 3.61 (s, 4H), 3.53 (m, 4H), 3.43 (m, 2H), 3.37-3.33 (m, 3H), 3.19-3.10 (m, 1H), 2.93-2.85 (m, 1H), 2.81-2.73 (m, 1H), 2.68 (d, 1H, J = 12.7 Hz), 2.18 (t, 2H, J = 7.4 Hz), 1.76-1.50 (m, 4H), 1.40 (q, 2H, J = 7.6 Hz).

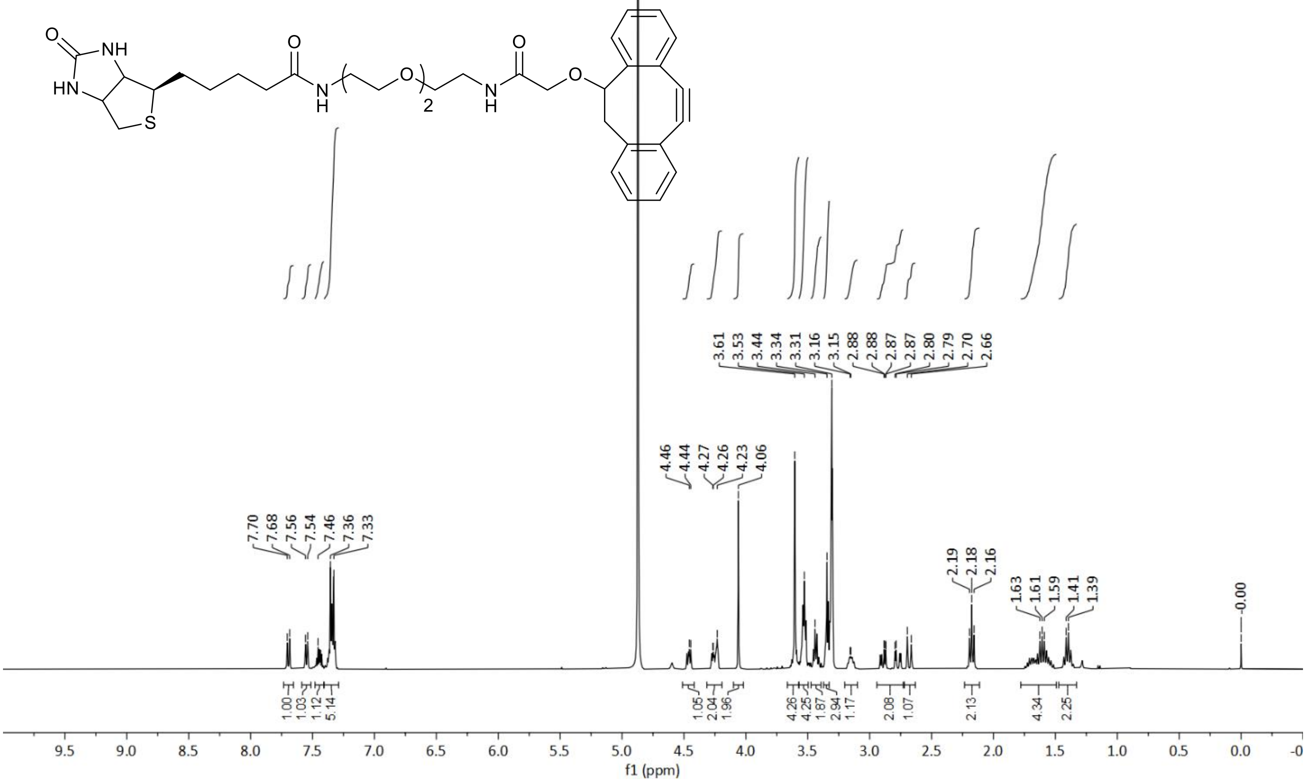

Fig. S16 ^{1}H NMR spectrum of Biotin-PEG$_2$-DIBO **10**

Fig. S17 ^{1}H NMR spectrum of Biotin-N_3 (13)

Biotin-N_3 (13)

^{1}H NMR (400 MHz, MeOH-d4): 4.52-4.45 (m, 1H), 4.33-4.26 (m, 1H), 3.35 (t, 2H, *J* = 6.7 Hz), 3.24 (t, 2H, *J* = 6.7 Hz), 3.24-3.16 (m, 1H), 2.96-2.88 (m, 1H), 2.70 (d, 1H, *J* = 12.7 Hz), 2.20 (t, 2H, *J* = 7.4 Hz), 1.80-1.53 (m, 6H), 1.44 (q, 2H, *J* = 7.7 Hz).

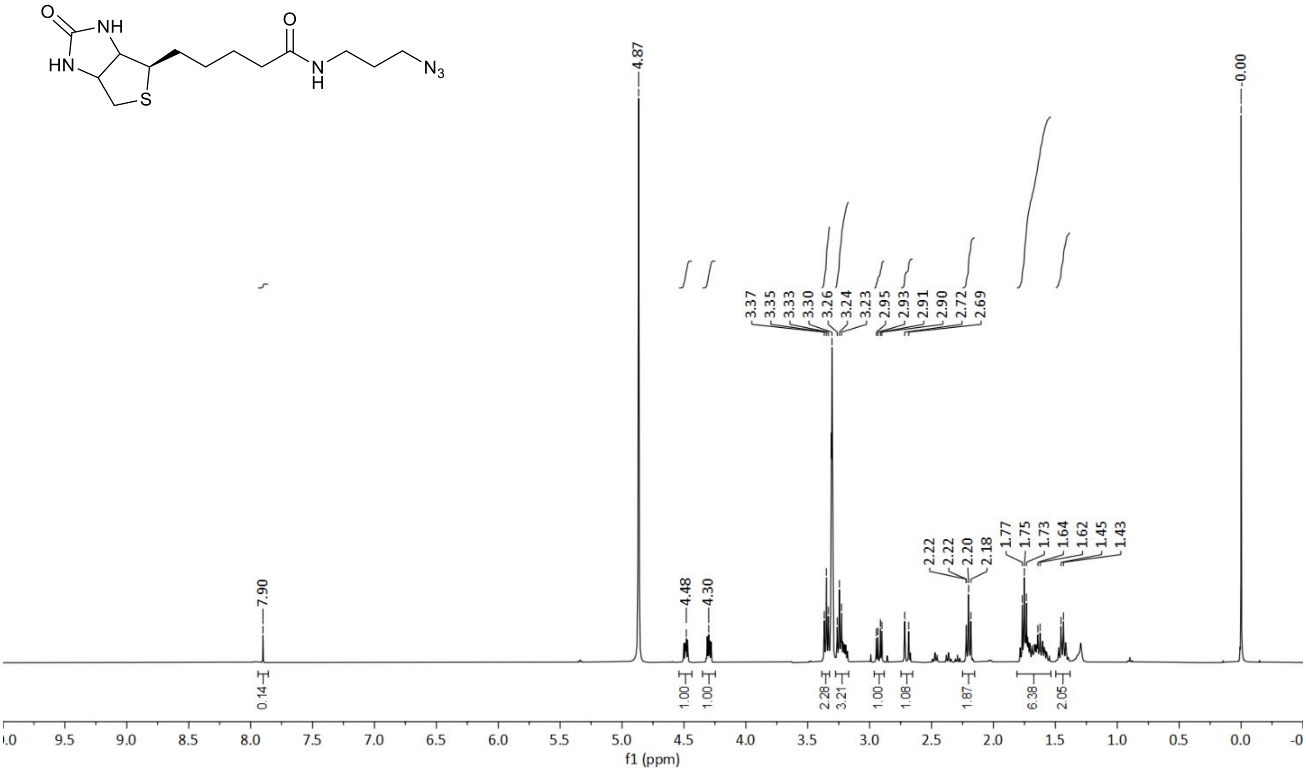

Fig. S17 ^{1}H NMR spectrum of Biotin-N_3 **13**

Fig. S18 ^{13}C NMR spectrum of Biotin-N_3 (13)

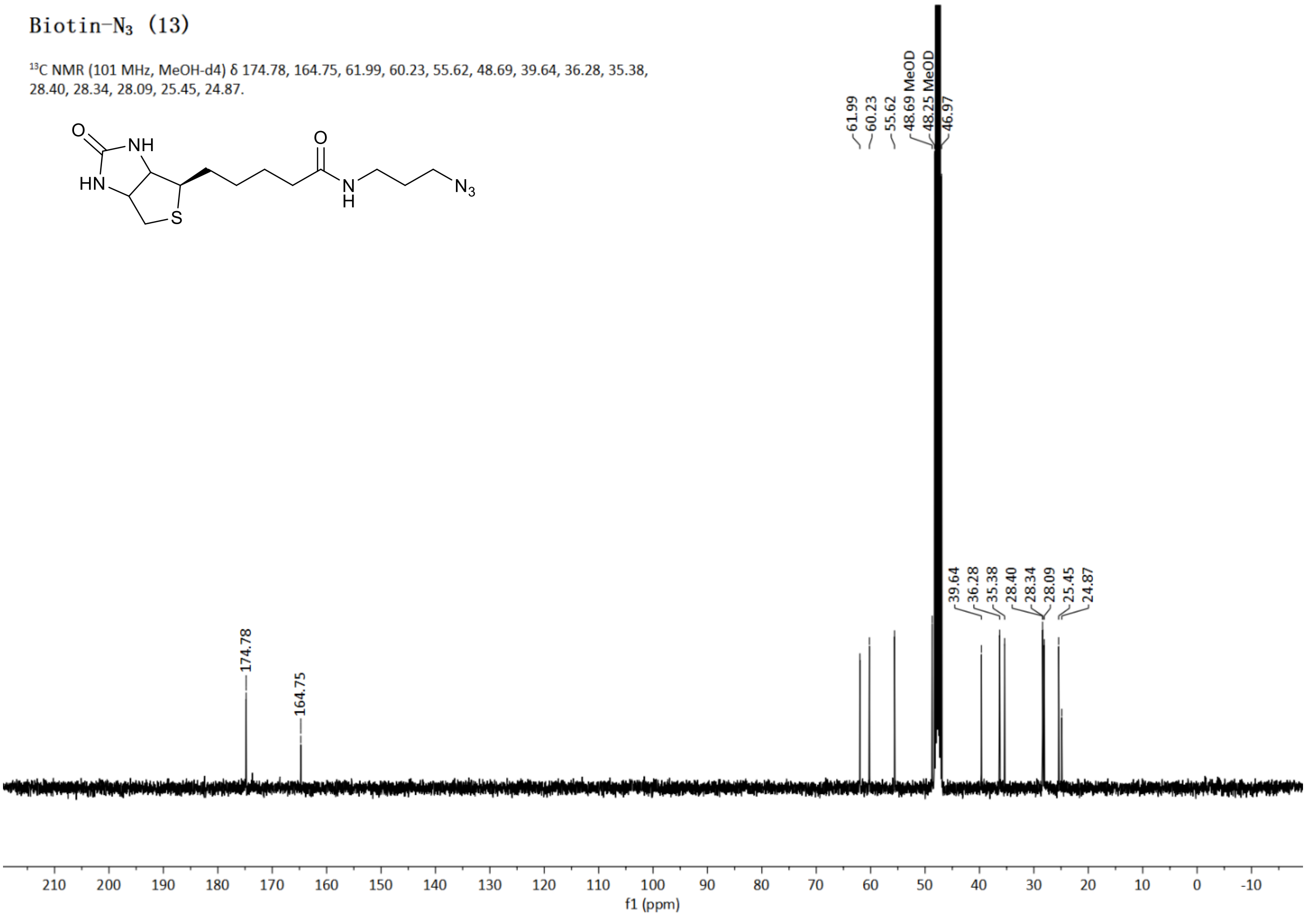

Fig. S18 ^{13}C NMR spectrum of Biotin-N_3 **13**

Supplementary References

1. F. C. Wu, H. Zhang, Q. Zhou, M. Wu, Z. Ballard, Y. Tian, J. Y. Wang, Z. W. Niu, Y. Huang, *Chemical communications (Cambridge, England)*, 2014, **50**, 4007.
2. C. Uttamapinant, A. Tangpeerachaikul, S. Grecian, S. Clarke, U. Singh, P. Slade, K. R. Gee, A. Y. Ting, *Angewandte Chemie International Edition*, 2012, **51**, 5852.
